# A Novel Therapeutic Mechanism of Imipridones ONC201/ONC206 in MYCN-Amplified Neuroblastoma Cells *via* Differential Expression of Tumorigenic Proteins

**DOI:** 10.3389/fped.2021.693145

**Published:** 2021-08-04

**Authors:** Sarra El-Soussi, Reine Hanna, Hanna Semaan, Amanda-Rose Khater, Jad Abdallah, Wassim Abou-Kheir, Tamara Abou-Antoun

**Affiliations:** ^1^Shool of Pharmacy, Lebanese American University, Byblos, Lebanon; ^2^Faculty of Sciences, Lebanese University, Fanar, Lebanon; ^3^Department of Anatomy, Cell Biology, and Physiological Sciences, Faculty of Medicine, American University of Beirut, Beirut, Lebanon

**Keywords:** ONC201/ONC206, Pdgfrβ, L1CAM, HMGA1, FABP5, cancer stem cells, MYCN-amplified neuroblastoma, γ-H2AX

## Abstract

Neuroblastoma is the most common extracranial nervous system tumor in children. It presents with a spectrum of clinical prognostic measures ranging from benign growths that regress spontaneously to highly malignant, treatment evasive tumors affiliated with increased mortality rates. MYCN amplification is commonly seen in high-risk neuroblastoma, rendering it highly malignant and recurrence prone. In our current study, we investigated the therapeutic potential of small molecule inducers of TRAIL, ONC201, and ONC206 in MYCN-amplified IMR-32 and non-MYCN-amplified SK-N-SH human neuroblastoma cell lines. Our results exhibit potent antitumor activity of ONC201 and ONC206 *via* a novel inhibition of EGF-induced L1CAM and PDGFRβ phosphorylation in both cell lines. Drug treatment significantly reduced cellular proliferation, viability, migration, invasion, tumorsphere formation potential, and increased apoptosis in both cell lines. The protein expression of tumorigenic NMYC, Sox-2, Oct-4, FABP5, and HMGA1 significantly decreased 48 h post-drug treatment, whereas cleaved PARP1/caspase-3 and γH2AX increased 72 h post-drug treatment, compared with vehicle-treated cells in the MYCN-amplified IMR-32 cell line. We are the first to report this novel differential protein expression after ONC201 or ONC206 treatment in human neuroblastoma cells, demonstrating an important multitarget effect which may yield added therapeutic benefits in treating this devastating childhood cancer.

## Introduction

Neuroblastoma (NB) is the most common solid extracranial pediatric tumor that arises from neural crest origin. It is the most common malignancy diagnosed in early infancy with 25–50 caser per million individuals (1). The median age at time of diagnosis is 19 months (2). Historically, NB is slightly more predominant in white children than in black children (3) with a higher male gender prevalence (4). Despite the relatively low incidence, NB accounts for almost 15% of all childhood cancer deaths, pointing out to the poor prognosis of the disease (5).

NB is often associated with characteristics of heterogeneous pathology and diverse clinical outcomes that range from spontaneous regression to rapid malignant progression (5, 6). While the initial treatment plan of NB depends on the patient's risk group, it usually ranges from observation only to intensive multimodal therapy including surgery, chemotherapy, and radiation. In addition to the conventional therapy options, there is a range of novel potential breakthrough therapies for NB among which are retinoic acid, targeted metaiodobenzylguanidine (MIBG) radiotherapy, antiangiogenic agents, and programmed cell-death 1/programmed cell-death 1 ligand (PD1/PDL1) inhibitors. Despite significant improvement in the treatment strategy of NB, high-risk patients still demonstrate <50% long-term survival (7) which warrants the need for more effective treatment strategies for this deadly disease.

**Figure 1:**
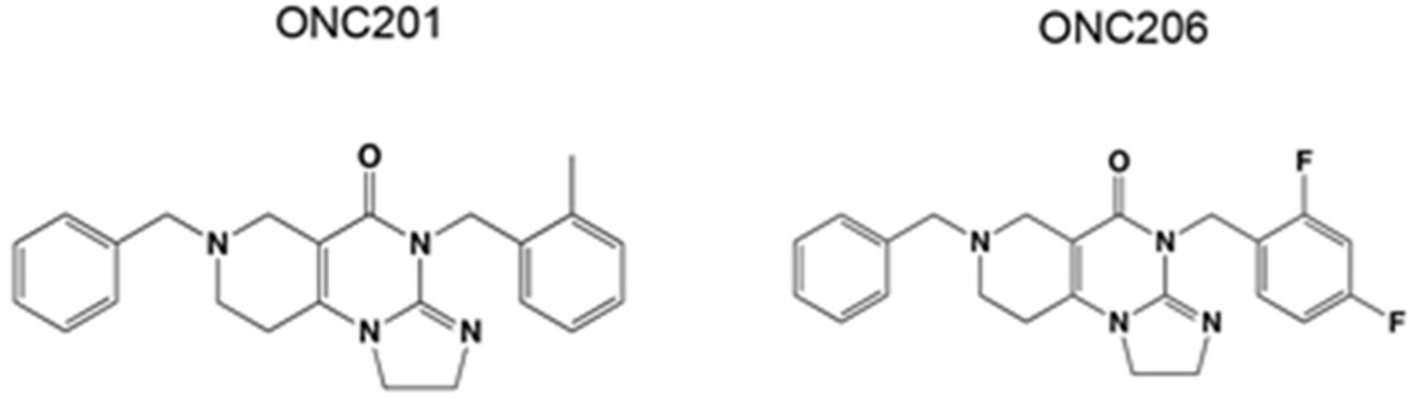
Chemical structure of ONC201 and ONC206.

ONC201 and its potent analog ONC206 are promising first in class small molecules which are characterized by their unique chemical structure (Figure 1). ONC201 was first identified in a phenotypic cell-based screen for selective p53-independent proapoptotic immune cytokine TNF-related apoptosis-inducing ligand (TRAIL) inducing compounds (8). Interestingly, the tumoricidal and apoptotic effects of ONC201 appear to be TRAIL dependent in solid tumors, whereas TRAIL independent in hematological tumors (9). Because of the favorable antitumor properties of ONC201, developing more potent analogs with enhanced efficacy was highly desirable. Thus, medicinal chemistry changes were applied to ONC201 while preserving its imidazopyridopyrimidone core structure to obtain ONC206. ONC206 is the most potent nanomolar DRD2 antagonist capable of activating ISR, while inhibiting Akt/ERK and inducing TRAIL/DR5. Its ability to increase tumor apoptotic rate while sparing healthy, non-cancerous cells makes it an ideal candidate for targeted cancer therapy (10–12).

In the TRAIL-dependent mechanism of action, ONC201 induces dual inactivation of AKT and ERK signaling pathways resulting in dephosphorylation of the transcription factor FOXO3a (Forkhead/winged-helix-box-class-O3), thus, leading to the upregulation of TRAIL expression (8). Moreover, ONC201 and its potent analog ONC206 were further reported to induce human mitochondrial caseinolytic protease P (ClpP) regulation and dopamine receptor DRD2 antagonism (13). On the other hand, in hematological tumors, ONC201 causes apoptotic effects by inducing prolonged integrated stress response (14). During such stress response, the transcription factor ATF4 is activated and subsequent expression of pro-apoptotic genes such as CHOP is induced (15). ONC201 is considered a novel anti-tumor candidate due to the unique features it possesses including: broad spectrum activity independent of tumor type or any existing mutations, oral bioavailability, ability to cross the blood brain barrier (BBB) and outstanding safety profile without toxicity on normal cells (16). ONC201 is currently in phase I/II clinical trials (13) as an antitumor therapeutic agent for several human cancers including neuroendocrine tumors and recurrent glioma (e.g., Clinicaltrials.gov NCT03034200). ONC206 has shown great promise in pre-clinical models including glioblastoma (10), high-grade gliomas, medulloblastomas, neuroblastomas, sarcomas (17), and serous endometrial cancer cells (18). The IND for ONC206 was accepted by the FDA and the first-in-human clinical trial (Clinicaltrials.gov NCT04541082) with a starting dose of 90 mg in biomarker-defined adult recurrent and rare central nervous system tumors was initiated (13) and is currently recruiting patients. Moreover, another clinical trial (Clinicaltrials.gov NCT04732065) due to initiate on June 30, 2021 will be recruiting patients with newly diagnosed or recurrent diffuse midline gliomas and other malignant brain tumors such as glioblastomas and spinal cord gliomas.

In our current work, we aimed to investigate the therapeutic effect of ONC201 and ONC206 in human pediatric NB cell lines including the MYCN-amplified IMR-32 and non-MYCN-amplified SK-N-SH cells. We report a significant reduction in cell proliferation, viability, migration/invasion, and tumorsphere formation, as well as significant upregulation of apoptosis after treatment with either drug. Moreover, we report a novel mechanism by which these small molecule imipridones exert their therapeutic effect including downregulation of highly tumorigenic proteins, upregulation of apoptotic markers, maintenance of phosphorylated H2A histone family member X (γ-H2AX) expression up to 72 h post-treatment, and a significant inhibition of the epidermal growth factor (EGF) triggered trans-activation of platelet-derived growth factor receptor-beta (PDGFRβ) and the L1 cell adhesion molecule (L1CAM). To the best of our knowledge, we are the first to report this novel differential tumorigenic protein expression and activation induced by these small-molecule imipridones and believe that this may open a new avenue for therapeutic investigation into these malignant childhood cancers that would possibly be useful considerations in clinical trials.

## Methods and Materials

### Cell Culture and Treatments

Neuroblastoma IMR-32 and SK-N-SH and medulloblastoma D556 and D283 (ATCC, Manassas, VA, USA) cells were cultured and maintained in Dulbecco's modified Eagle's media (DMEM; cat. No. D6429, Sigma-Aldrich, St. Louis, MO, USA) including 4,500 mg/L glucose, l-glutamine, sodium pyruvate, and supplemented with 10% heat inactivated fetal bovine serum (FBS; cat. No. F9665, Sigma-Aldrich) and 1% penicillin/streptomycin (cat. No. P4333, Sigma-Aldrich). Cells were incubated at 37°C in a humidified incubator containing 5% CO_2_. The drugs ONC201 and ONC206 were graciously provided by Oncoceutics Inc. (Philadelphia, PA, USA) on a material transfer agreement (MTA) and were both reconstituted in dimethyl sulfoxide (DMSO; cat. No. D2650, Sigma-Aldrich), per manufacturer's instructions.

### Cell Proliferation Assay/WST-1

Cells were seeded (1.5 × 10^3^ cells/well for IMR-32 and 3 × 10^3^ for SK-N-SH) in 50 μl complete medium in 96-well plates and incubated overnight. After 24 h, the vehicle wells were treated with 0.01% of DMSO in 50 μl of complete media. The drug-treated wells were incubated with increasing concentrations of either ONC201 or ONC206 to the respective wells with 50 μl of complete medium for the entire duration of the experiment. At each time point, 10 μl/well of the water-soluble tetrazolium salt-1 (WST-1; cat. No. ab155902; Abcam, Cambridge, UK) yellow dye is added to the cells and incubated for 3 h. The absorbance was detected after 3 h using a Synergy HTX Multi-Mode Microplate Reader at 450 nm (BioTek, Winooski, VT, USA). Each experiment was performed in triplicates to assess for consistency of results. The data was collected, and the absorbance values were graphed to analyze the dose and time effect of ONC201/ONC206 on cellular proliferation.

### “Wound-Healing” Assay

IMR-32 cells were cultured in 12-well plates (2 × 10^5^ cells/well) and incubated overnight until they reached 90–100% confluence. Cells were then treated with 10 mg/ml of mitomycin C (Sigma) for 30 min in order to block cellular proliferation. A sterile 200 μl tip was used to create a uniform wound through the cell monolayer. The cells were then washed, treated respectively with either vehicle, 5 μM ONC201 or 0.5 μM ONC206 for the entire duration of the experiment and cultured in 5% FBS media. Images from eight random fields were taken at 0 h immediately after “wound induction” and 24 h “post-wound-induction.” “Wound healing” was analyzed using AxioVision LE Application by subtracting the area of the wound at 24 h from the area of the wound at 0 h. The average area of “wound closure” was calculated from all the acquired images and graphed using GraphPad Prism.

### EGF Stimulated Wound-Healing Assay

IMR-32 and SK-N-SH cells were cultured in 24-well plates (1.5 × 10^5^ cells/well) and incubated overnight until they reached 90–100% confluence. Cells were then starved with serum free media in order to block cellular proliferation and deplete present growth factors. After 24 h of starvation, a sterile 200 μl tip was used to create a uniform wound through the cell monolayer. The cells were then washed, treated respectively with either vehicle, 5 μM ONC201 or 0.5 μM ONC206 for the entire duration of the experiment and cultured in serum free media with or without 200 ng of EGF. Images were taken at 0 and 24 h by which the distance traveled by the cells enumerated the closure of the wounds.

### EGF-Stimulated Trans-Well Migration or Invasion Assay

IMR-32 cells were grown to ~90–100% confluence and then serum starved overnight with either vehicle or ONC206 (1 μM) treatment. The next day, cells were harvested, counted, and 5 × 10^5^ cells were suspended in serum-free media containing either vehicle or ONC201/ONC206 and added to the upper compartment of an ECM-coated trans-well invasion (Millipore cat. No. ECM550; Burlington, MA, USA) or collagen-coated trans-well migration (Millipore cat. No. ECM508) chamber and allowed to migrate for 24 h or invade for 48 h to the lower compartment of the chamber that was submerged in either serum-free media or serum-free media supplemented with 200 ng EGF. The EGF-induced cellular migration/invasion was determined by the number of cells that invaded through the ECM-coated chamber to the underside of the well. Cell stain dye (Crystal violet) was added for 10 min and subsequently extracted using a dye extraction solution and the absorption of the dye was detected on a standard microplate reader (560 nm). Cell stain was used to visualize the invaded cells prior to stain extraction and quantitation. Absorbance was measured at 560 nm using Synergy HTX Multi-Mode microplate reader. The absorbance obtained from all the vehicle-treated wells was normalized to “1,” and all treatment groups were calculated as a relative “fold change” of the vehicle-treated cells.

### Trypan Blue Exclusion (Cellular Viability) Assay

The cytotoxicity of ONC201 and ONC206 was evaluated using trypan blue (cat. No. T8154; Sigma) as an exclusion dye in order to determine the number of viable and dead cells in a cell suspension; 25 × 10^3^ IMR-32 and SK-N-SH cells were plated in each well of a 12-well plate to a final volume of 1 ml/well. After an overnight incubation to allow cell attachment, the media was replaced and cells were incubated for 48 and 72 h with complete media with vehicle or drug to a final concentration of 5 μM ONC 201 or 0.5 μM ONC 206 for the entire duration of the experiment. Following each time point, the cells were collected and a volume of each condition was mixed with an equal volume of trypan blue for a 1:1 (V/V) dilution. Ten microliters of this mixture was placed on a Neubauer improved cell counting chamber and examined under an optical microscope. Viable (white) and dead (blue) cells were counted in four outer squares of the hematocytometer, and the average was calculated.

### Apoptosis Assay

IMR-32 and SK-N-SH cells were seeded in a 24-well plate with a density of 0.25 × 10^5^ cells/well. After 24 h, the cells were treated with a final concentration of either 5 μM ONC 201 or 0.5 μM ONC 206 and compared with vehicle-treated control cells for the entire duration of the experiment. At 72 h after drug treatment, the media was removed and replaced with 200 μl of 10% FBS DMEM containing the CellEvent Caspase-3/7 green reagent (cat. No. C10423; ThermoFisher Scientific) at a final concentration of 2 μM. After 30 minutes of incubation, random fields were imaged to capture the fluorescent cells using a Zeiss Axio Observer Microscope (Zeiss LSM 900). Fluorescent and total cells were counted and the percentage of fluorescent cells was calculated.

### Protein Interactions Analysis

Protein interactions were studied using STRING database (https://string-db.org/). This database serves to highlight functional enrichments as well as protein associations imported from other databases of curated biological pathway knowledge such as Gene Ontology (GO) and Kyoto Encyclopedia of Genes and Genomes (KEGG), which were used in our study to define molecular functions as well as interactions between the proteins of interest. These interactions include direct (physical) and indirect (functional) associations. The names of the proteins were entered into the database, and a diagram of interactions was generated along with tables containing functions of these proteins, some of which were chosen to be displayed in a color-coded manner in our diagrams.

### Western Blotting

IMR-32 and SK-N-SH cells were cultured in six-well plates (0.3 × 10^6^ cells/well) and incubated overnight. After 24 h, the media was collected and replaced with either the vehicle or the drug at the final concentration of 5 μM ONC 201 or 0.5 μM ONC 206 for the entire duration of the experiment. The cells were collected by scraping at 48 and 72 h for protein extraction. Cell lysates were mixed with 2 × Laemmli buffer (cat. No. S3401; Sigma) used for denaturation and loading of protein samples. Total protein (20 μg) from every treatment group was loaded onto a TGX stain-free fast cast 10% SDS-PAGE gel (cat. No. 161-0183; Bio-Rad, Hercules, CA, USA), electrophoresed at 120 V for 1 h and transferred onto a PVDF membrane (cat. No. 162-0177; Bio-Rad). The membrane was blocked with 3% BSA (A2153; Sigma) and then incubated with 1:1,000 dilution of primary antibody against L1CAM (rabbit pAb ab123990; Abcam), p-L1CAM (rabbit pAb ab61009; Abcam), MycN (rabbit mAb 84406; Cell Signaling Technology, Danvers, MA, USA), Oct-4 (rabbit mAb 2840S; Cell Signaling Technology), Sox-2 (rabbit mAb 2748; Cell Signaling Technology), FABP5 (rabbit mAb 39926S; Cell Signaling Technology), HMGA1 (rabbit mAb 7777S; Cell Signaling Technology), p-ERK (sc-7383; Santa Cruz Biotechnology, Dallas, TX, USA), ERK1/2 (rabbit mAb 4695S; Cell Signaling Technology), p-AKT (rabbit mAb 4060; Cell Signaling Technology), AKT (rabbit mAb 4685; Cell Signaling Technology), p-PDGFRβ (rabbit pAb, ab16868; Abcam), PDGFRβ (rabbit pAb ab32570; Abcam), cleaved-caspase 3 (rabbit mAb 9661; Cell Signaling Technology), total-caspase 3 (rabbit mAb 9662; Cell Signaling Technology), cleaved-PARP1 (rabbit mAb 5625; Cell Signaling Technology), total-PARP1 (rabbit mAb 9532; Cell Signaling Technology), γH2AX (rabbit mAb 5438; Cell Signaling Technology), and H2AX (rabbit mAb 7631; Cell Signaling Technology) overnight at 4°C. The membrane was then washed with TBST and incubated with a secondary peroxidase-conjugated antibody (cat. No. 170-5046; Bio-Rad) for 1 h at room temperature. The membrane was then washed with TBST, incubated in Clarity ECL substrate (cat. No. 170-5061; Bio-Rad) and quantified by densitometric analysis using Image Lab software from Bio-Rad Laboratories. Stain-free blot normalization was used to normalize the bands of interest relative to the total protein in that lane instead of a housekeeping gene as a loading control.

### EGF-Induced Transctivation of L1CAM and PDGFRβ

IMR-32 and SK-N-SH cells were cultured in six-well plates (0.3 × 10^6^ cells/well) and incubated overnight. After incubation for 24 h, the media was collected and replaced with either the vehicle or the drug at the final concentration of 5 μM ONC 201 or 0.5 μM ONC 206 for the entire duration of the experiment. After 24 h of treatment, the cells were stimulated with 50 ng of EGF for 15 min. The cells were collected by scraping for protein extraction. Cell lysates were mixed with 2× Laemmli buffer (cat. No. S3401; Sigma) used for denaturation and loading of protein samples. Total protein (20 μg) from every treatment group was loaded onto a TGX stain-free fast cast 10% SDS-PAGE gel (cat. No. 161-0183; Bio-Rad), electrophoresed at 120 V for 1 h, and transferred onto a PVDF membrane (cat. No. 162-0177; Bio-Rad). The membrane was blocked with 3% BSA (A2153; Sigma) and then incubated with 1:1,000 dilution of primary antibody overnight at 4°C. The membrane was then washed with TBST and incubated with a secondary peroxidase-conjugated antibody (cat. No. 170-5046; Bio-Rad) for 1 h at room temperature. The membrane was then washed with TBST, incubated in Clarity ECL substrate (cat. No. 170-5061; Bio-Rad) and quantified by densitometric analysis using Image Lab software from Bio-Rad Laboratories. Stain-free blot normalization was used to normalize the bands of interest relative to the total protein in that lane instead of a housekeeping gene as a loading control.

### 3D Culture and Sphere-Formation Assay

Cells (2,000 cells/well) were suspended in Matrigel™/serum free media (1:1 dilution). The growth factor-reduced Matrigel™ (cat. No. 354230; BD Biosciences, Franklin Lakes, NJ, USA) was thawed on ice at 4°C overnight prior to its use. The solution was plated gently around the rims of the wells of a 24-well plate (50 μl per well). The Matrigel was then allowed to solidify for 45 min in the incubator at 37°C. Meanwhile, serum-free media was supplemented with 5% FBS and 5 μg/ml of Plasmocin prophylactic (cat. code ant-mpp; InvivoGen, San Diego, CA, USA) used to prevent mycoplasma contamination. For the treatment with ONC201 and ONC206, this media was supplemented additionally with increasing concentrations of the drug at 2.5/5 μM ONC 201 or 0.5/1 μM ONC 206 for the entire duration of the experiment. Afterwards, 500 μl/well of the prepared media was added to the center of the wells accordingly, and it was regularly changed every 2–3 days. At day 8 after plating, images of the IMR-32 and SK-N-SH spheres were taken in order to measure the diameter of the sphere diameter *via* Zeiss software. In addition, the number of spheres in each well was counted, and the sphere formation efficiency (SFE) was calculated using the following formula: SFE = (number of spheres counted/number of seeded cells) × 100.

### Statistical Analysis

All experiments were conducted in triplicate wells/plates (three experimental triplicates) and repeated three independent times on different days and using different cell passages for a total of three biological replicates. The means ± the standard error of the means (SEM) of all independent repeats were calculated and the statistical analysis performed using GraphPad Prism Software. A Two-way ANOVA was used for viability and WST-1, whereas one-way ANOVA was used for apoptosis, wound-healing and trans-well invasion/migration assays and Western blot analysis of protein expression between drug and vehicle-treated cells. Statistical significance was set as *p*-value < 0.05.

## Results

### ONC201/206 Significantly Inhibited Cellular Viability and Proliferation

**Figure 2:**
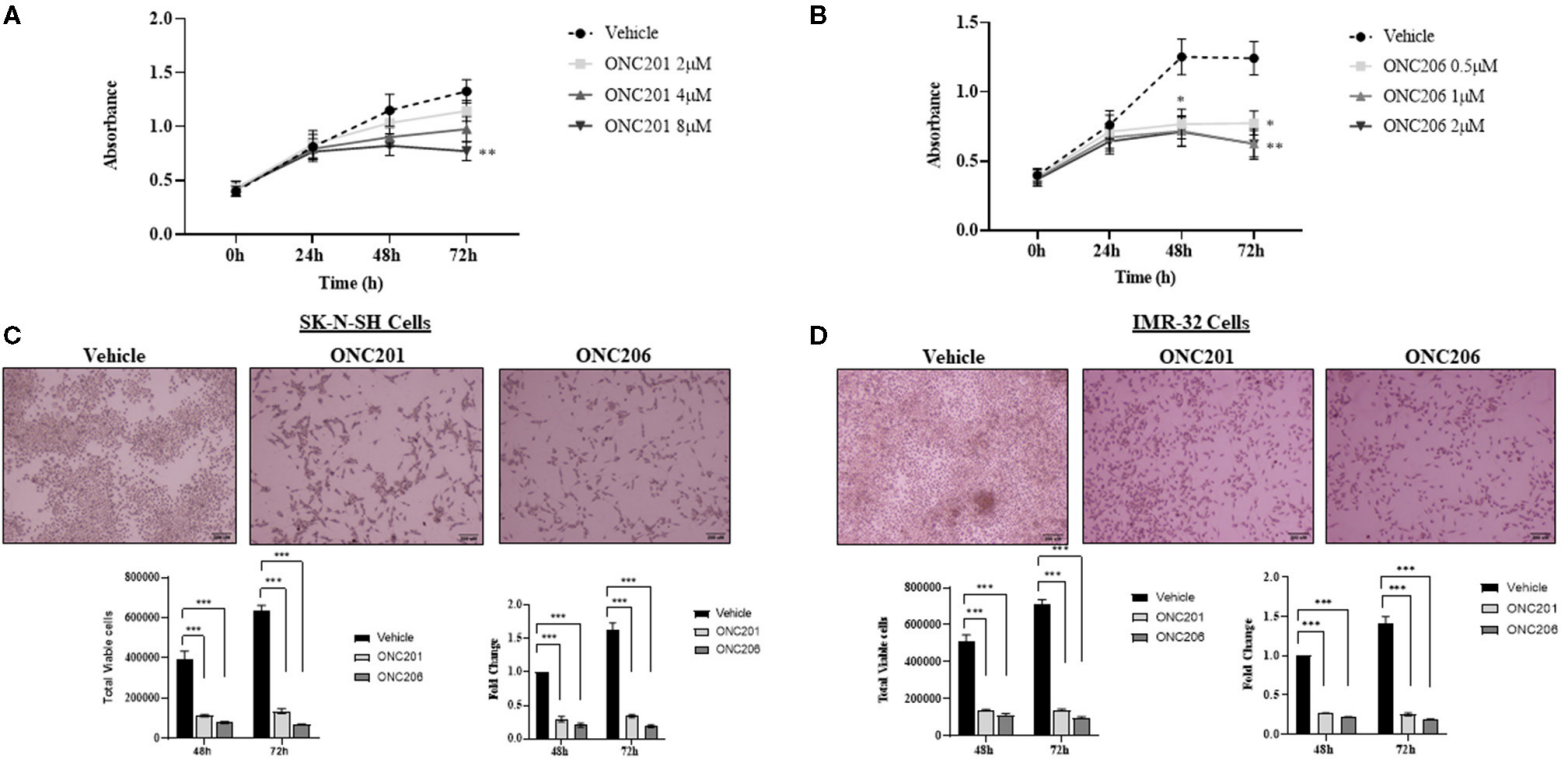
Cellular proliferation and viability inhibited with ONC201/ONC206 treatment. IMR-32 cells treated with increasing doses of either ONC201 **(A)** or ONC206 **(B)** exhibited a dose- and time-dependent decrease in cellular proliferation compared with vehicle-treated cells. SK-N-SH **(C)** and IMR-32 **(D)** cells treated with either ONC201 (5 μM) or ONC206 (0.5 μM) exhibited a significant reduction in cellular viability at 72 h post-treatment. Experiments were conducted in triplicates and repeated three times. Data represent the mean ± the standard error of the mean (SEM) of multiple experiments. *p < 0.05; **p < 0.01; ***p < 0.001.

IMR-32 cells treated with either ONC201 or ONC206 exhibited significant dose- and time-dependent reduction in cellular proliferation as assessed using the WST-1 assay (Figures 2A,B). Treatment of SK-N-SH and IMR-32 cells with either ONC201 (5 μM) or ONC206 (0.5 μM) significantly reduced cellular viability at 48 and 72 h post-treatment (Figures 2C,D). ONC206 was 10× more potent than ONC201 in reducing cellular proliferation and viability in our cells. Cellular viability and proliferation was also significantly reduces after treatment with either ONC201 or ONC206 in human neuroblastoma SH-SY5Y (Supplementary Figure 1) and human medulloblastoma D556 (Supplementary Figures 2, 4) and D283 cells (Supplementary Figure 3) compared with vehicle-treated counterparts.

### ONC201/206 Significantly Inhibited Cellular Migration and Invasion

**Figure 3:**
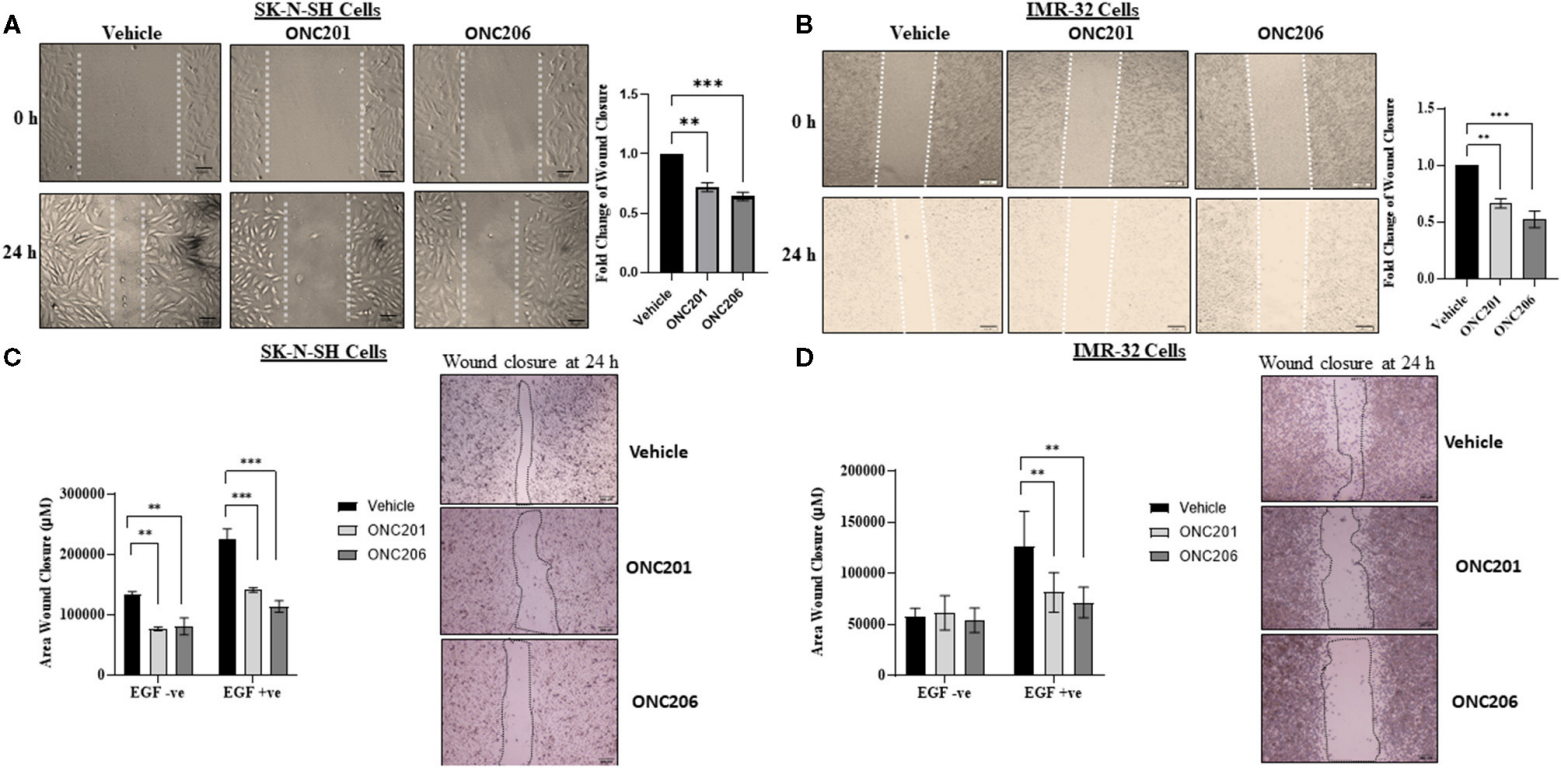
Cellular migration inhibited with ONC201/ONC206 treatment. SK-N-SH **(A)** and IMR-32 **(B)** cells treated with either ONC201 (5 μM) or ONC206 (0.5 μM) exhibited a significant reduction in cellular migration compared with vehicle-treated cells. SK-N-SH **(C)** and IMR-32 **(D)** cells treated with either ONC201 (5 μM) or ONC206 (0.5 μM) exhibited a significant reduction in EGF-induced cellular migration as assessed using the wound-healing assay. Experiments were conducted in triplicates and repeated three times. Data represent the mean ± the standard error of the mean (SEM) of multiple experiments. **p < 0.01; ***p < 0.001.

**Figure 4:**
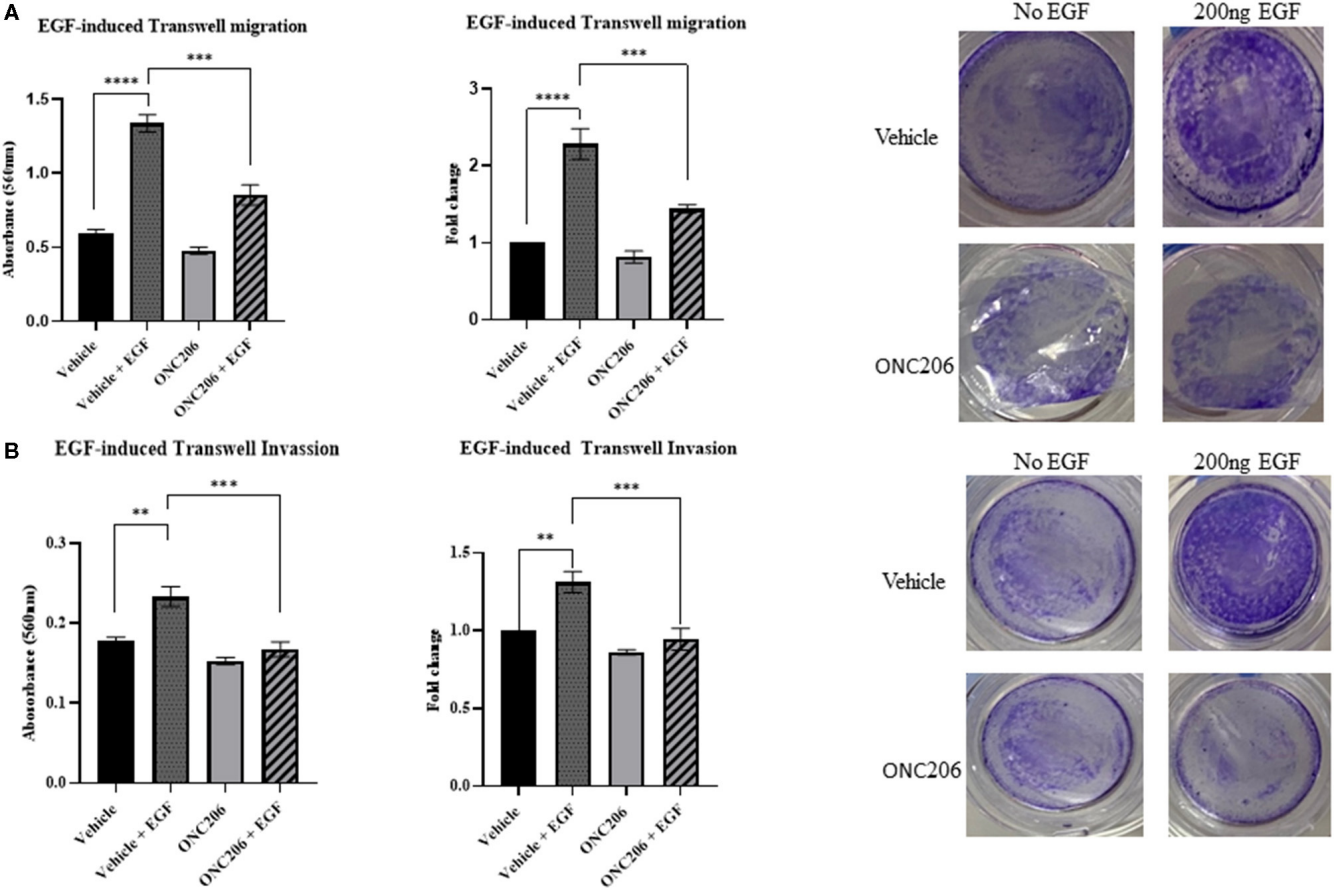
EGF-induced cellular trans-well migration and invasion inhibited with ONC206 treatment. IMR-32 cells treated with ONC206 (1 μM) exhibited a significant reduction in the EGF-induced cellular migration **(A)** and invasion **(B)** through the ECM-coated trans-well chambers compared with vehicle-treated cells. Experiments were conducted in triplicates and repeated three times. Data represent the mean ± the standard error of the mean (SEM) of multiple experiments. **p < 0.01; ***p < 0.001; ****p < 0.0001.

SK-N-SH and IMR-32 cells treated with ONC201 (5 μM) or ONC206 (0.5 μM) exhibited significant reduction in cellular migration as assessed using a “wound-healing” scratch assay (Figures 3A,B). Cells were treated with drugs and serum starved overnight and pre-treated the next day with 10 mg/ml mitomycin C for 30 min before wound induction and then allowed to migrate and close the wound in response to 5% FBS for 24 h. The cellular ability to migrate into the wound and close it was significantly decreased in ONC201 and ONC206 treated, compared with vehicle-treated cells. Next, we assessed the EGF-induced cellular migration capacity 24 h post-wound induction in drug-treated, serum-starved cells. Treatment with either drug significantly reduced the EGF-induced ability of SK-N-SH (Figure 3C) and IMR-32 (Figure 3D) to migrate into and close the wound (Figures 3C,D). Trans-well cellular migration through a collagen coated chamber (Figure 4A) and invasion through an ECM-coated chamber (Figure 4B) was assessed in IMR-32 cells with or without ONC206 (1 μM) treatment. Serum-starved and drug-treated (overnight) cells were given 24 h to migrate through the collagen-coated or 48 h to invade through the ECM-coated trans-well chamber toward the lower well containing serum-free media or serum-free media supplemented with 200 ng EGF. ONC206 treatment overnight significantly inhibited the cellular ability to migrate or invade through the trans-well chamber to the bottom side of the membrane.

### ONC201/206 Significantly Increased Cellular Apoptosis

**Figure 5:**
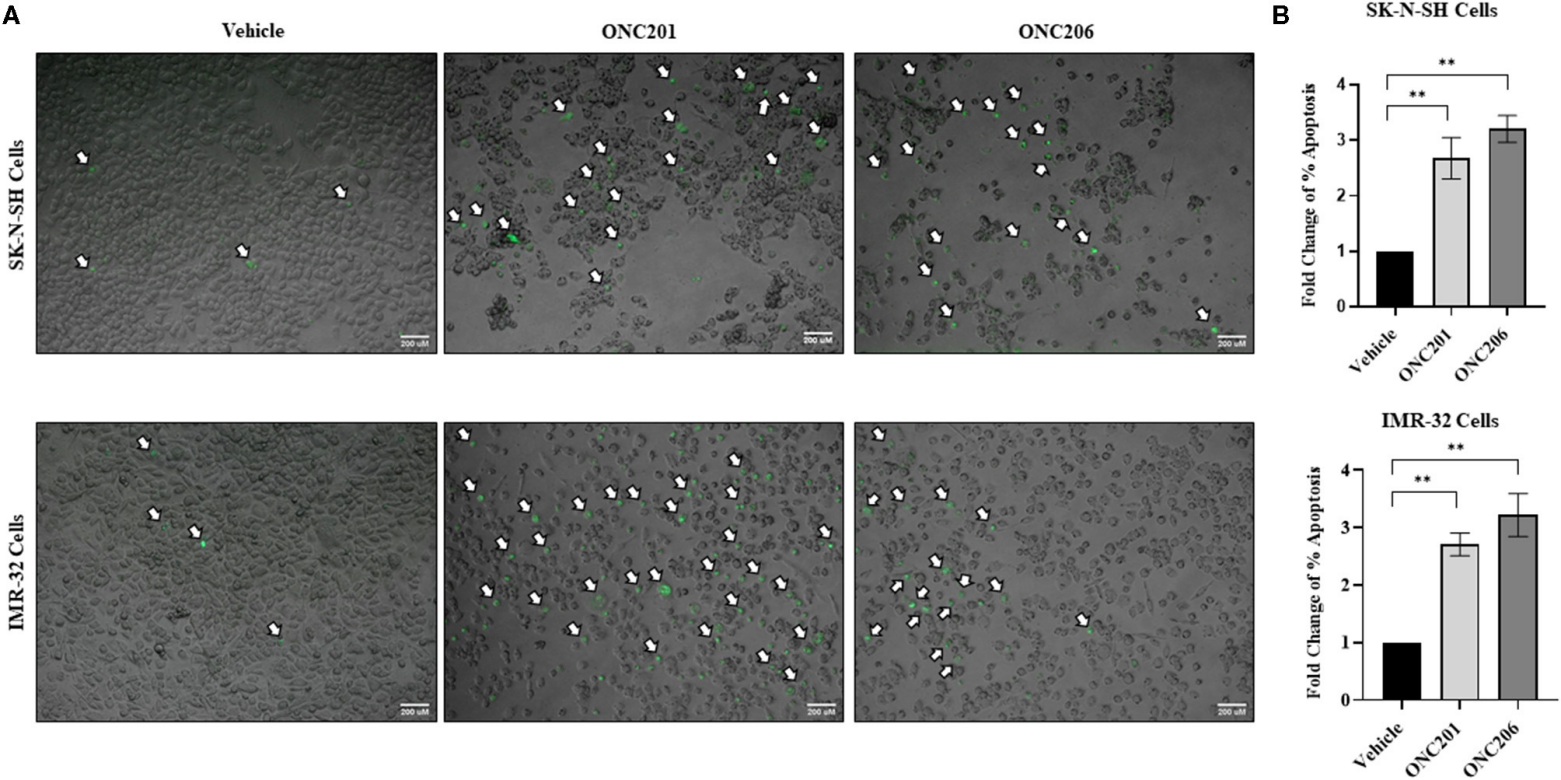
Cellular apoptosis after ONC201/ONC206 treatment. SK-N-SH and IMR-32 cells treated with ONC201 (5 μM) or ONC206 (0.5 μM) exhibited a significant increase in the rate of cellular apoptosis as assessed using the CellEvent caspase3/7 staining 72 h post-treatment. Green fluorescence represents apoptotic cells imaged at ×4 magnification **(A)** and relative fold change of percent apoptosis compared with vehicle-treated cells **(B)**. White arrows indicate fluorescent apoptotic cells. Experiments were conducted in triplicates and repeated three times. Scale bar 200 μM. Data represent mean ± standard error of the mean (SEM) of multiple experiments. **p < 0.01.

SK-N-SH and IMR-32 cells treated with either ONC201 or ONC206 exhibited significant induction of apoptosis at 72 h post-treatment (Figures 5A,B). A single dose of ONC201 (5 μM) or ONC206 (0.5 μM) treatment in either cell line was sufficient to significantly increase the rate of cellular apoptosis 72 h post-treatment compared with vehicle-treated cells as evidenced by increased fluorescence of CellEvent caspase 3/7 staining (white arrows) of the cells (Figure 5A) and by quantitative analysis of the ratio of apoptotic cells relative to the entire cell population (Figure 5B) and graphed as a fold change of the vehicle-treated cells. Specifically, SK-N-SH cells exhibited 31 and 41% apoptosis in ONC201- and ONC206-treated cells, respectively compared with only 11.5% in vehicle-treated cells. Moreover, IMR-32 exhibited 35 and 40% apoptosis in ONC201 and ONC206 treated cells, respectively compared with only 12% in vehicle-treated cells. Similarly, medulloblastoma D556 and D283 cells exhibited significant induction of apoptosis 72 h after treatment with either ONC201 or ONC206 compared with vehicle-treated cells (Supplementary Figure 5).

### ONC201/206 Significantly Inhibited Tumorsphere Formation Efficiency

**Figure 6:**
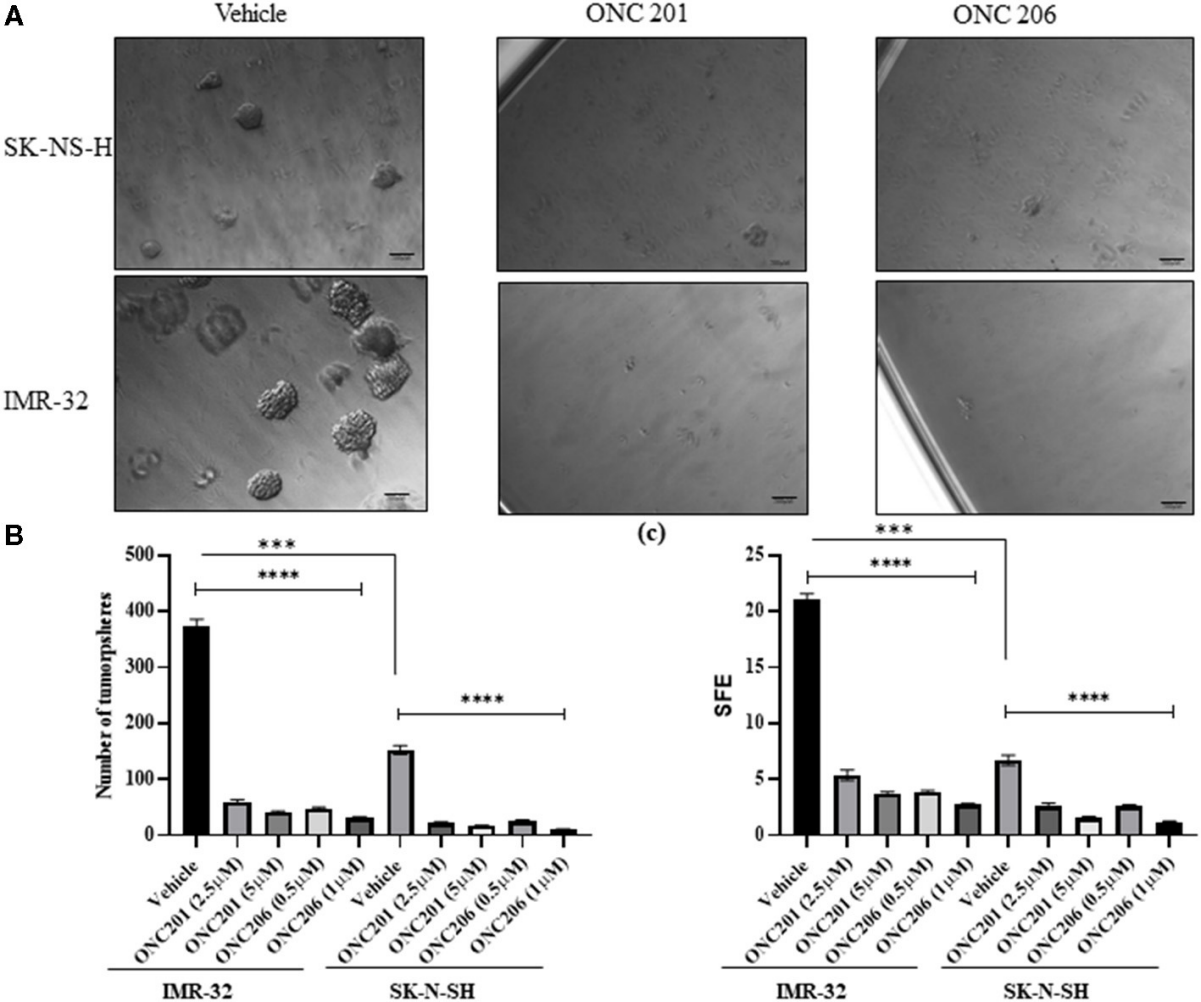
Stem-cell tumorsphere self-renewal potential after ONC201/ONC206 treatment. SK-N-SH and IMR-32 cells treated with ONC201 (2.5 or 5 μM) or ONC206 (0.5 or 1 μM) exhibited a significant inhibition in the tumorsphere formation potential compared with vehicle-treated cells as assessed using the sphere formation efficiency (SFE) assay. IMR-32 cells exhibited a statistically significant higher rate of SFE compared with SK-N-SH cells. The representative micrographic images **(A)**, sphere numbers **(B)**, and SFE **(C)** illustrate the significant reduction in tumorsphere formation potential in both cell lines after drug treatment. Experiments were conducted in triplicates and repeated three times. Data represent the mean ± the standard error of the mean (SEM) of multiple experiments. ****p < 0.0001. ***Statistical significance <0.001.

SK-N-SH and IMR-32 cells treated with either ONC201 or ONC206 exhibited significant dose-dependent reduction in tumorsphere formation potential compared with vehicle-treated cells (Figure 6). Stem-cell proliferation capacity as assessed using the tumorsphere formation assay of cells grown in stem cell media supplemented with EGF and FGF, was significantly inhibited in both IMR-32 and SK-N-SH cells treated with either ONC201 (2.5 and 5 μM) or ONC206 (0.5 and 1 μM). The SFE revealed that, while IMR-32 cells (Figure 6A lower panel) exhibited a significantly higher rate of sphere formation potential compared with SK-N-SH cells (Figure 6A upper panel), giving rise to significantly more tumorspheres with larger diameters, both cell lines exhibited significant inhibition of SFE 8 days after a single dose of either drug and at both the low and high concentrations (Figures 6B,C).

### ONC201/206 Induced Differential Expression of Tumorigenic Proteins

**Figure 7:**
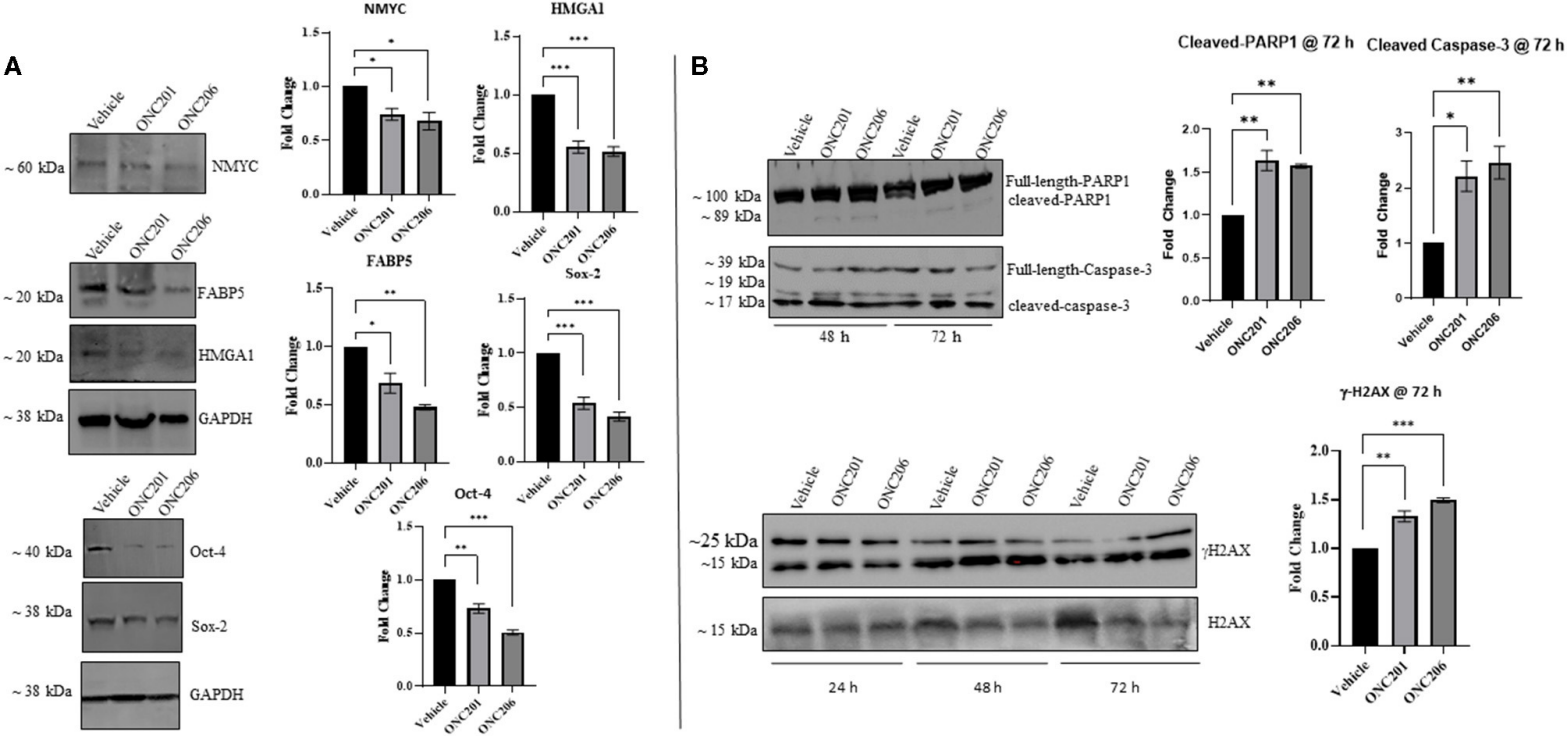
ONC201/ONC206 treatment induces differential expression of tumorigenic proteins in IMR-32 NB cells. IMR-32 cells treated with either ONC201 (5 μM) or ONC206 (0.5 μM) exhibited significant reduction of tumorigenic protein expression compared with vehicle-treated cells as assessed using Western blot analyses. **(A)** Western blot quantitative analysis revealed significant downregulation in the protein expression of tumorigenic NMYC, FABP5, and HMGA1 as well as stem-markers Oct-4 and Sox-2, 48 h after drug treatment. **(B)** Cleaved caspase-3, cleaved-PARP1, and γH2AX were upregulated at 48 and 72 h post-drug treatment. Blots without GAPDH loading control were quantitated using Bio-Rad's stain-free gel technology and normalized to the total protein loaded per lane. Experiments were conducted in triplicates and repeated three times. Data represent the mean ± the standard error of the mean (SEM) of multiple experiments. *p < 0.05; **p < 0.01; ***p < 0.001.

In order to elucidate the molecular mechanisms behind the anticancer efficacy of the single dose of imipridones ONC201 and ONC206, we characterized, *via* Western blot analyses, the protein expression of various tumorigenic markers at 48 and 72 h post-treatment. Interestingly, the protein expression of the oncogenic NMYC, the tumorigenic fatty-acid-binding protein 5 (FABP5), and the high mobility group A1 (HMGA1) was significantly reduced 48 h after treatment with either ONC201 (5 μM) or ONC206 (0.5 μM) compared with vehicle-treated MYCN-amplified IMR-32 cells. In addition, the protein expression of stem cell markers Oct-4 and Sox-2 was also reduced at 48 h post-drug treatment (Figure 7A).

In addition, a single dose of either ONC201 or ONC206 led to sustained phosphorylated γ-H2AX expression and increased cleaved PARP1 and cleaved-caspase-3 at 48 and 72 h compared with vehicle-treated cells (Figure 7B). Of note is the substantial potency of ONC206 (0.5 μM) compared with ONC201 (5 μM) which revealed similar or greater differential expression of tumorigenic proteins compared with ONC201 at a dose 10× lower than that used for ONC201.

**Figure 8:**
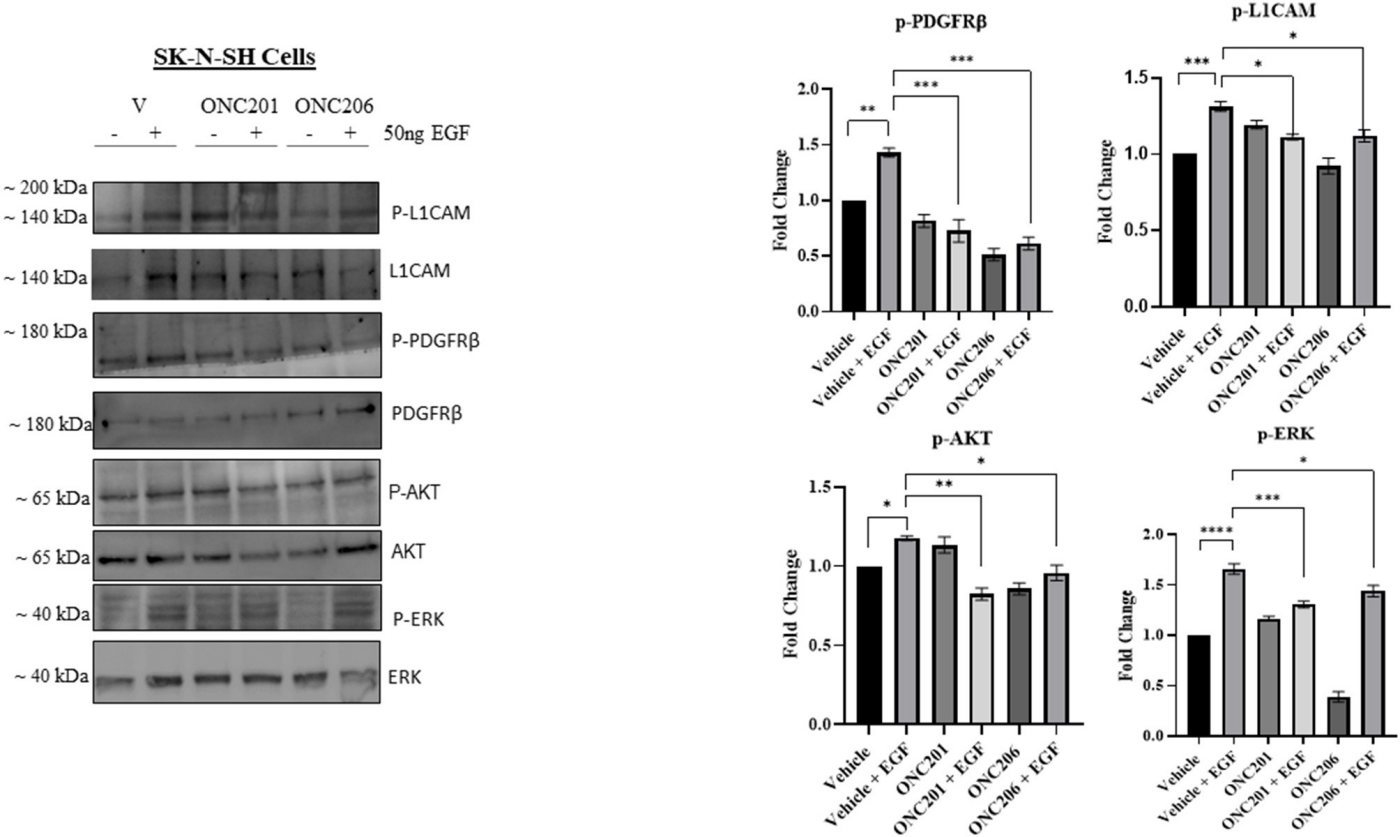
ONC201/ONC206 treatment induces differential expression of tumorigenic proteins in SK-N-SH cells. EGF-induced phosphorylation of L1-CAM, PDGFRβ, and AKT/ERK was significantly reduced with a single continuous dose of either ONC201 (5 μM) or ONC206 (0.5 μM) treatment compared with vehicle-treated cells. Experiments were conducted in triplicates and repeated three times. Data represent the mean ± the standard error of the mean (SEM) of multiple experiments. *p < 0.05; **p < 0.01; ***p < 0.001; ****p < 0.0001.

**Figure 9:**
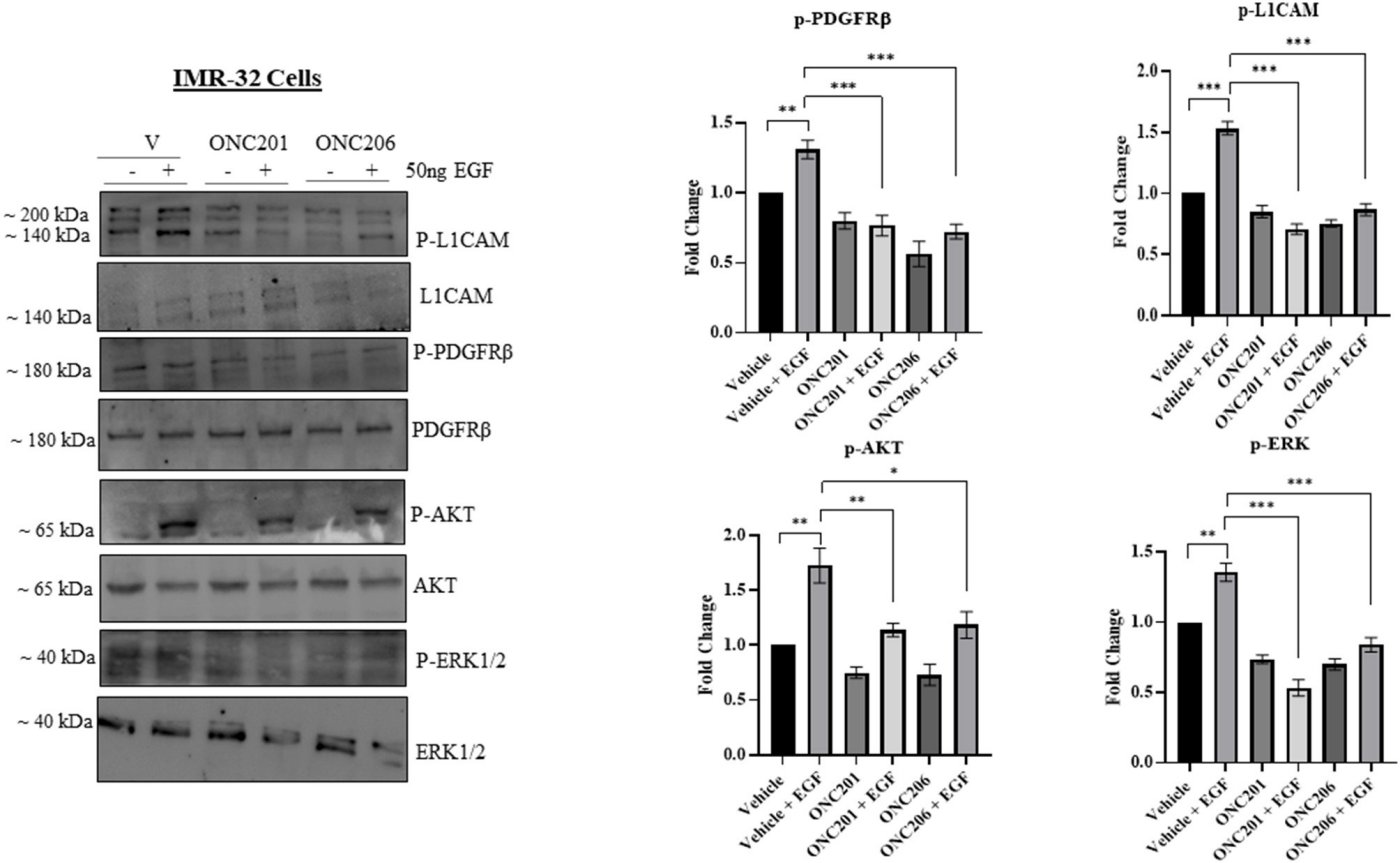
ONC201/ONC206 treatment induces differential expression of tumorigenic proteins in IMR-32 cells. EGF-induced phosphorylation of L1-CAM, PDGFRβ, and AKT/ERK was significantly reduced with a single continuous dose of either ONC201 (5 μM) or ONC206 (0.5 μM) treatment compared with vehicle-treated cells. Experiments were conducted in triplicates and repeated three times. Data represent the mean ± the standard error of the mean (SEM) of multiple experiments. *p < 0.05; **p < 0.01; ***p < 0.001.

In order to delineate any possible interplay between tumorigenic pathways in our cells in the face of drug therapy, we serum starved cells overnight and pre-treated them with either drug for 1 h before triggering EGF-induced phosphorylation of target proteins. Interestingly, we observed an EGF-induced trans-activation of PDGFRβ and L1CAM with subsequent phosphorylation of AKT/ERK that was effectively inhibited with either ONC201 or ONC206, but more potently with ONC206 treatment compared with vehicle-treated SK-N-SH (Figure 8) and IMR-32 (Figure 9) cells. This points to an interesting inhibitory effect of these drugs on tumorigenic pathways that interconnect and communicate in an attempt to drive therapeutic resistance and malignancy.

## Discussion

**Figure 10:**
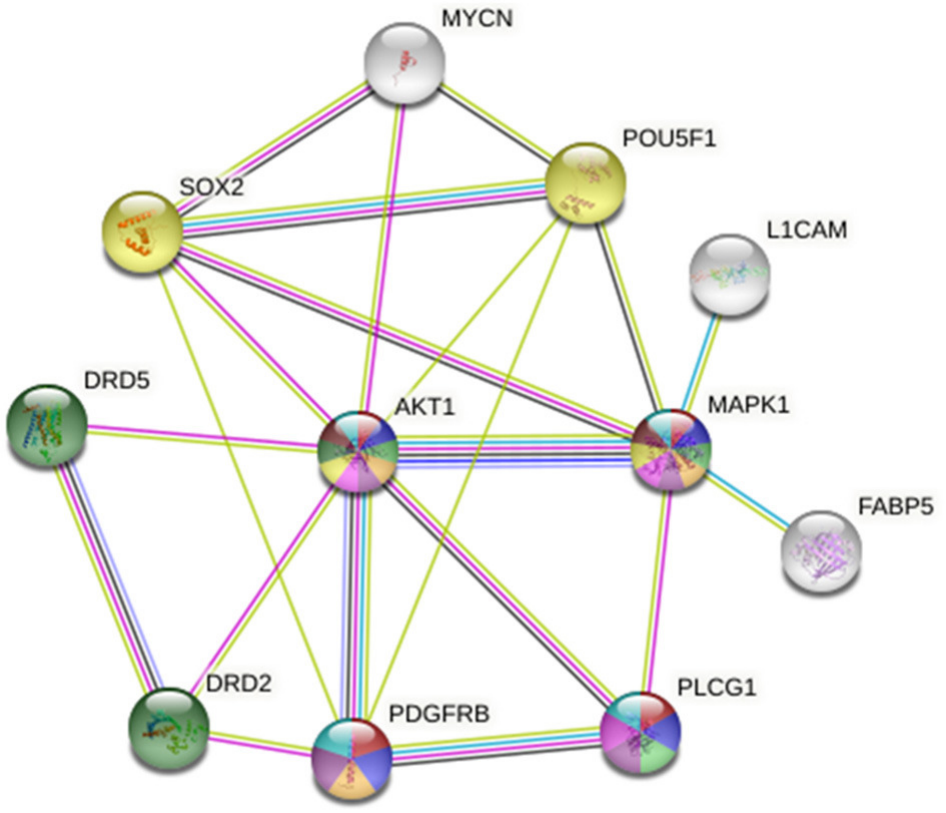
STRING protein network analysis reveals an intricate interplay between the inhibitory targets of ONC201 and ONC206, DRD2/DRD5, and AKT/MAPK1, and other tumorigenic proteins implicated in cellular mechanisms of cancer progression, metastasis, and drug resistance. This protein interaction visualization demonstrates the complex interplay between AKT, MAPK, and many other proteins including PDGFRβ, Sox-2, Oct-4, MYCN, FABP5, and L1CAM that play key roles in tumor initiation, progression, metastasis, recurrence, and cancer stem-cell maintenance. The sources of these interactions are derived from experimental results (pink lines), curated databases (blue lines), gene co-expression (black lines), gene co-occurrence (dark blue lines), and text mining (green lines).

Neuroblastoma that presents with MYCN amplification is highly malignant and treatment evasive, increasing the rate of tumor recurrence and mortality in children afflicted with this devastating disease. It is of utmost importance to deliver highly targeted and effective therapy to such cancers in order to achieve better cures and reduce the possibility of malignant, treatment-resistant recurrence. The imipridones ONC201 and ONC206 from Oncoceutics Inc. have shown promise in various cancers including recurrent and metastatic neuroendocrine tumors (Clinicaltrials.gov NCT03034200) and aggressive pediatric brain tumors such as glioblastomas and high-grade gliomas *via* TRAIL induction along with ClpP activation and DRD2 inhibition (13, 19). By activating C1pP and deregulating mitochondrial integrity and triggering the integrated stress response, ONC201 and ONC206 induce cellular apoptosis in a TRAIL-dependent manner (15, 20). In our current work, we reveal a novel mechanism of therapeutic efficacy utilized by ONC201 and ONC206 to induce antitumor activity in malignant neuroblastoma cells. Specifically, the drugs significantly inhibited cellular proliferation, viability, migration, invasion, tumorsphere formation potential and increased cellular apoptosis. Mechanistically, we observed that the two small-molecule imipridones sustained γH2AX and increased cleaved-PARP1/caspase-3 expression by 72 h post-treatment and significantly downregulated the protein expression of tumorigenic FABP5, HMGA1, NMYC, and stem cell pluripotency markers Sox-2 and Oct-4. String protein network and KEGG pathway analysis mapped these proteins along with ONC targets (DRD2 and DRD5) to highly tumorigenic pathways (see Figure 10 and Table 1). Moreover, we identified a unique EGF-induced trans-activation of L1CAM and PDGFRβ that was significantly inhibited with either ONC201 or ONC206 treatment but more potently with ONC206 yielding similar effects, but at 0.5 μM concentration compared with 10× that concentration using ONC201. We previously reported on the PDGFRB-induced transactivation of EGFR in medulloblastoma (21, 22) and neuroblastoma (23) cells that was effectively abrogated with imatinib and sunitinib. In this current work, we showed a novel EGF-induced transactivation of L1CAM and PDGFRβ that was effectively abrogated with either ONC201 or ONC206 treatment. This is a novel finding and warrants further investigation to delineate whether the effect of the drugs is mediated through inhibition of the EGFR pathway or through another tumorigenic pathway that is yet to be determined.

**Table 1:** KEGG pathway analysis reveals the top tumorigenic pathways in which our proteins are affiliated indicating the count in network, strength, and the false-discovery rate.

**Pathway**	**Description**	**Count in network**	**Strength**	**False discovery rate**
hsa01521	EGFR tyrosine kinase inhibitor resistance	5 of 78	2.02	1.28e-07
hsa05230	Central carbon metabolism in cancer	4 of 65	2.0	3.13e-06
hsa05214	Glioma	4 of 68	1.98	3.13e-06
hsa05218	Melanoma	4 of 72	1.96	3.13e-06
hsa05213	Endometrial cancer	3 of 58	1.93	4.47e-05
hsa04370	VEGF signaling pathway	3 of 59	1.92	4.47e-05
hsa05219	Bladder cancer	2 of 41	1.9	0.00095
hsa05210	Colorectal cancer	4 of 85	1.88	3.13e-06
hsa05223	Non-small cell lung cancer	3 of 66	1.87	5.74e-05
hsa01524	Platinum drug resistance	3 of 70	1.84	6.36e-05
hsa05214	Prostate cancer	4 of 97	1.83	3.98e-06
hsa05231	Choline metabolism in cancer	4 of 98	1.82	3.98e-06
hsa05212	Pancreatic cancer	3 of 74	1.82	7.16e-05
hsa04012	ErbB singaling Pathway	3 of 83	1.77	9.59e-05
hsa04540	Gap junctions	3 of 87	1.75	0.00011
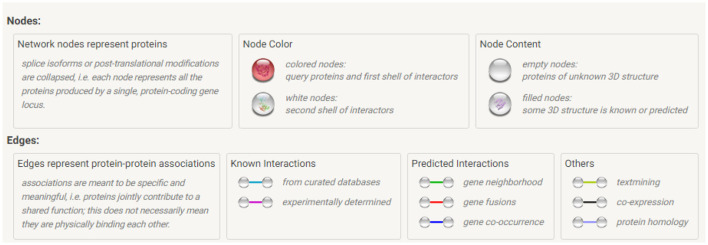				

While neuroblastomas are not intracranial brain tumors, rather are extracranial nervous system tumors of the periphery, they do however share molecular signatures with pediatric intracranial brain tumors. For example, resistance to TRAIL-induced apoptosis (24), EGFR and MYCN amplification (25), along with PDGFR activation (26), overexpression of stem-cell markers Oct-4 and Sox-2 (27) and epigenetic deregulation (28) have been reported in medulloblastomas. Moreover, the small-molecule imipridones were highly effective against H3 K27M-mutant diffuse midline glioma in adults and children. Neuroblastomas have also been reported to harbor epigenetic deregulation involving H3K27 (29) that led to epigenetic silencing of tumor suppressor genes, and MYCN-amplified neuroblastomas were further found to effectively respond to H3K27 demethylation inhibition (30) using GSK-J4. Whether ONC201 and ONC206 are inducing an epigenetic-directed therapeutic effect on our cells is yet to be determined and one of our main goals for future directions in our research projects.

FABP5 is a well-established carrier for long-chain fatty acids and retinoic acid transporter responsible for regulating the delivery of retinoic acid to the nuclear receptor peroxisome proliferator-activated receptor delta, which subsequently induces cellular proliferation and survival. Li et al. (31) reported the statistically significant upregulation of FABP5 in highly proliferative, recurrence prone craniopharyngiomas. Barbus et al. (32) reported the statistically significant upregulation of FABP5 mRNA in tumors of short-term (<6 months) compared with long-term (>36 months) survivors of glioblastoma, the expression of which was associated with increased proliferation in the tumors and activation of 3-phosphoinositide-dependent protein kinase-1, indicating the tumorigenic involvement of FABP5 in this highly malignant and lethal brain cancer. A single dose of either ONC201 (5 μM) or ONC206 (0.5 μM) significantly downregulated FABP5 expression 48 h post-treatment, which may elude to an interesting therapeutic avenue for these drugs that warrants further investigation. This ONC-induced downregulation of FABP5 may explain one of the mechanisms that led to the significant reduction in cellular proliferation and viability after drug treatment compared with vehicle-treated cells in our model.

HMGA1 belongs to the family of high mobility group proteins that is responsible for governing global chromatin remodeling (33). HMGA1 binds to A/T-rich DNA sequences in gene promoter and enhancer regions, antagonizing the linker histone H1 and inducing an open chromatin structure thereby facilitating gene transcription (34). Its function is critical in highly proliferative cells during embryonic growth, development, and cellular differentiation, a time when it is highly expressed after which its expression recedes to almost undetectable levels in fully differentiated, non-proliferative cells (35). Interestingly, HMGA1 expression becomes upregulated in a vast majority of tumors such as glioblastomas (36), neuroblastomas (37), and many other solid tumors, including breast (38), pancreatic (39), cervical (40), thyroid (41), prostate (42), colon (43), and renal cell carcinoma (44) and has been highly affiliated with poor prognosis and tumor metastasis (45). To the best of our knowledge, we are the first to demonstrate this ONC201/ONC206-induced inhibition of the HMGA1 protein expression, which may shed some interesting light onto a novel mechanism by which these small molecule imipridones may be inducing their therapeutic efficacy in our model.

ONC201 is the first bitopic DRD2 antagonist in clinical oncology (46). DRD2 involvement in cancer stem cell maintenance and proliferation was previously reported (47). The authors demonstrate that STAT3/IL6 stimulation governed the DRD2-induced stem cell self-renewal in triple-negative breast cancers, while the induction of cell-cycle arrest at G1 and reduction in cell proliferation and viability were reported to be independent of DRD2. Another report supports the tumorigenic role of DRD2 in cancer stem cells, by demonstrating that DRD2 is a downstream target of the repressor element-1 silencing transcription factor (REST) that drives tumorigenesis in glioblastoma stem-like cells by regulating invasion and apoptosis (48). STRING protein network analysis revealed a pivotal interaction between DRD2, DRD5 and AKT, which serves as a hub that connects PDGFRβ, Sox-2, Oct-4 (POU5F1), MYCN, MAPK1, PLCG1, L1CAM, and FABP5 (Figure 10). This intricate network of connections between these highly tumorigenic and cancer stem-cell maintenance proteins drives cellular proliferation, migration/invasion, tumor progression, treatment-evasion, and cancer stem-cell self-renewal potential.

The KEGG pathway analysis places these protein interactions in highly tumorigenic and drug-resistance pathways. The top 15 pathways identified by STRING in which these proteins play crucial roles include (1) EGFR tyrosine kinase inhibitor resistance, (2) central carbon metabolism in cancer, (3) glioma, (4) melanoma, (5) endometrial cancer, (6) VEGF signaling pathway, (7) bladder cancer, (8) colorectal cancer, (9) non-small-cell lung cancer, (10) platinum drug resistance, (11) prostate cancer, (12) choline metabolism in cancer, (13) pancreatic cancer, (14) ErbB signaling pathway, and (15) gap junctions (Table 1). For the complete list of pathways, please see Supplementary Table 1. The ability of these two small-molecule imipridones to dysregulate and inhibit the activation of this vast number of tumorigenic proteins is therapeutically advantageous and opens a very exciting avenue for further investigation in other tumors that depend on these pathways to thrive.

Our studies demonstrate an interesting interconnection between tumorigenic pathways, namely, EGFR, PDGFR, and L1CAM. We revealed a significant EGF-induced transactivation of PDGFRβ and L1CAM that was potently inhibited with a single dose of either ONC201 or ONC206 pre-treatment in NB cells. The PDGFRβ plays pivotal roles in the regulation of embryonic development, cellular proliferation, survival, differentiation, migration, invasion, and chemotaxis (49, 50). L1CAM is a well-established neural cell adhesion molecule that was reported to drive cancer stem cell self-renewal potential, tumor survival, and evasion of apoptosis in gliomas (51). Moreover, it was recently reported to become highly overexpressed in the cerebrospinal fluid of patients with glioblastoma and brain cancer metastatic lesions compared with control patients with no tumors, indicating its potential role in promoting the development of glioblastoma and the metastasis and invasion of solid tumors into the brain (52). We previously reported the significant upregulation of L1CAM in the MYCN-amplified IMR-32 cells compared with the non-MYCN-amplified SK-N-SH counterparts. Moreover, we reported the irradiation-induced upregulation of L1CAM in NB IMR-32 cells and specifically in the cancer-stem-like cell subpopulation within the bulk tumor (53, 54). ONC201 and more potently ONC206 significantly reduced the EGF-induced trans-activation of L1CAM and PDGFRβ by inhibiting their EGF-induced phosphorylation. This inhibition may explain in part the reduced tumorsphere formation potential as evidenced by a significant reduction in SFE capacity in our cells as well as the significant reduction in cellular proliferation, viability and migration/invasion. The increased potential for the IMR32 cells to form tumorspheres in stem cell media compared with the SK-N-SH cells (Figure 6) may be explained by their significantly higher expression of L1CAM and MYCN relative the SK-N-SH cells. In fact the, SFE of the IMR-32 cells was a ~20%, whereas that of the SK-N-SH cells was only ~7%. The ability of a drug to eradicate the SFE of highly malignant cells and reduce the activation and expression of stem-cell maintenance proteins would serve as a promising compound that can eliminate the bulk tumor cells along with the malignant and recurrence prone subpopulation of stem cells within the bulk tumor.

To determine the proapoptotic mechanism of ONC201 and ONC206 in our cells, we examined the level of expression of cleaved-caspase 3 and cleaved-PARP1 and found an upregulation of both at 48 and 72 h after a single dose of either drug in the MYCN-amplified IMR-32 cells compared with the vehicle-treated controls. Moreover, we assessed the level of phosphorylated H2AX expression up to 72 h post-treatment with either drug and found a significant upregulation of the γH2AX expression over time relative to vehicle-treated controls. The increased cleaved-caspase 3 and cleaved-PARP1 expression in cancer cells indicates their inability to repair DNA excisions (55) thereby undergoing apoptosis. This is the proposed mechanism by which treatment with either ONC201 or ONC206 in our cells induced apoptosis. By crippling their ability to repair DNA, cells eventually succumbed to this therapeutic insult and underwent apoptosis. This is further supported by the persistent upregulation of γ-H2AX up to 72 h post-treatment further implicating the defective DNA repair mechanisms after drug treatment in our highly malignant, MYCN amplified IMR-32 NB cells.

NMYC amplification in NB has been implicated in resistance to apoptotic pathways (56, 57) including resistant to TRAIL-induced apoptosis (58) where previous studies have demonstrated the synergistic proapoptotic effect of combined TRAIL treatment in MYCN-negative neuroblastoma cells expressing caspase-8 following IFN-γ treatment (58). In our model, ONC201 and ONC206 effectively induced apoptosis in both cell lines, with ONC206 exhibiting a more potent effect with a dose 10× lower than that of ONC201. The Western blot analysis revealed an effective ability of either drug to inhibit NMYC expression in the MYCN-amplified IMR-32 cells, thereby possibly explaining the pro-apoptotic efficacy of these drugs on this malignant cell line. In future direction, we aim to elucidate the expression of TRAIL targets and other proteins governing these pathways after treatment with either drug in order to hone in on the precise mechanisms by which these drugs induce their pro-apoptotic effects in our model.

Future pre-clinical models and subsequent clinical trials would benefit from our current findings to focus on the relevant tumorigenic pathways that may be ideal candidates for these small molecule imipridones. Moreover, the novel tumorigenic targets identified in our work would be invaluable in guiding the design of combinatorial pre-clinical animal models to delineate the *in vivo* therapeutic efficacy of ONC 201 and ONC 206.

## Conclusion

While brain cancers in children differ vastly from those diagnosed in the adult population, there are some redundancies in the molecular drivers expressed in both (59) including PDGFR, AKT, and MAPK signaling. Moreover, MYCN and EGFR amplification, PDGFR activation, resistance to TRAIL-induced apoptosis, epigenetic oncogenic deregulation, and increased expression of stem-cell maintenance markers are features shared by both the intracranial brain tumors such as medulloblastoma and the extracranial peripheral nervous system tumor, neuroblastoma. We report herein the effective inhibition and downregulation of some of these players in our model using either ONC201 or ONC206. We have identified a unique and novel mechanism of therapeutic efficacy using these small molecule imipridones in malignant MYCN-amplified neuroblastoma cells.

They effectively abrogated the expression and activation of key tumorigenic proteins in highly aggressive, cancer-promoting pathways. These proteins were identified to be intricately affiliated either directly or indirectly with each other and with the primary targets of ONC201/ONC206 (DRD2/DRD5 and AKT/MAPK1). By breaking this delicate network of interactions between these malignancy-driving, treatment-resistance, and recurrence-inducing proteins, the small molecule imipridones serve as highly promising therapeutic molecules in treating the most aggressive cancers. Further studies are warranted to elucidate the effects of combinatorial therapy using these compounds with chemo- or radiotherapy to determine if a synergistic effect of dual therapy exists. Moreover, our findings report the effects of these drugs in an *in vitro* model, yet it is crucial to delineate these therapeutic effects in an *in vivo* pre-clinical model. In future directions, we aim to investigate the therapeutic efficacy of these drugs on a panel of pediatric tumors with differential molecular signatures and MYCN-amplification status in order to delineate their mechanism of action at the molecular, genetic, and epigenetic levels. Moreover, we aim to investigate the *in vivo* therapeutic efficacy of these drugs in combination with standard-of-care therapy and other small molecule, targeted therapy on tumorigenicity, invasion and metastasis, and overall survival using a murine animal model.

## Data Availability Statement

The original contributions generated for the study are included in the article/Supplementary Material, further inquiries can be directed to the corresponding author.

## Author Contributions

WA-K and TA-A: conceptualization, formal analysis, and resources. SE-S, RH, A-RK, and HS: data curation. JA and TA-A: funding acquisition and software. SE-S and HS: investigation. SE-S, RH, A-RK, HS, and TA-A: methodology. JA, WA-K, and TA-A: project administration. RH, WA-K, and TA-A: supervision. A-RK, HS, and WA-K: validation. TA-A: writing (original draft). SE-S, RH, A-RK, HS, JA, and WA-K: writing (review and editing). All authors contributed to the article and approved the submitted version.

## Conflict of Interest

The authors declare that the research was conducted in the absence of any commercial or financial relationships that could be construed as a potential conflict of interest.

## Publisher's Note

All claims expressed in this article are solely those of the authors and do not necessarily represent those of their affiliated organizations, or those of the publisher, the editors and the reviewers. Any product that may be evaluated in this article, or claim that may be made by its manufacturer, is not guaranteed or endorsed by the publisher.
