# Supplementary figures and images for: A Novel Therapeutic Mechanism of Imipridones ONC201/ONC206 in MYCN-Amplified Neuroblastoma Cells via Differential Expression of Tumorigenic Proteins
Source: Front Pediatr. 2021 Aug 4;9:693145. doi: 10.3389/fped.2021.693145 (PMC8373200; doi:10.3389/fped.2021.693145)

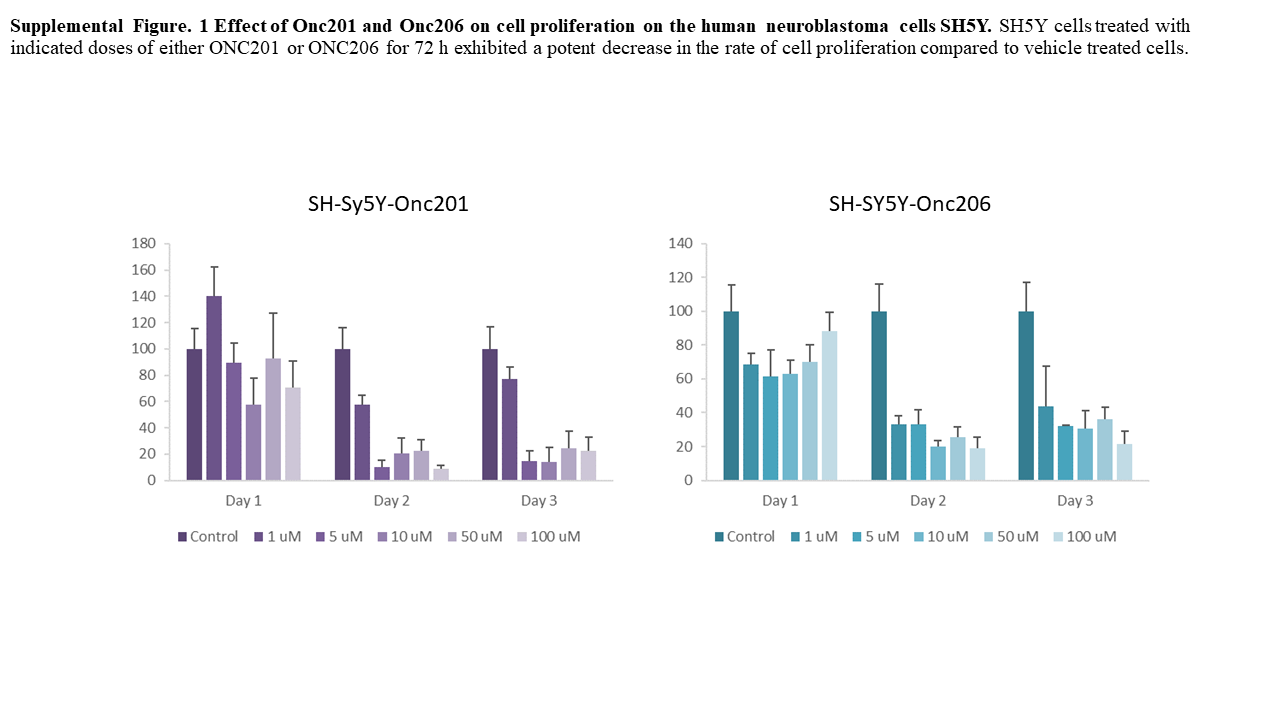

Supplement: Supplementary file 1 [file Image_1.TIF]

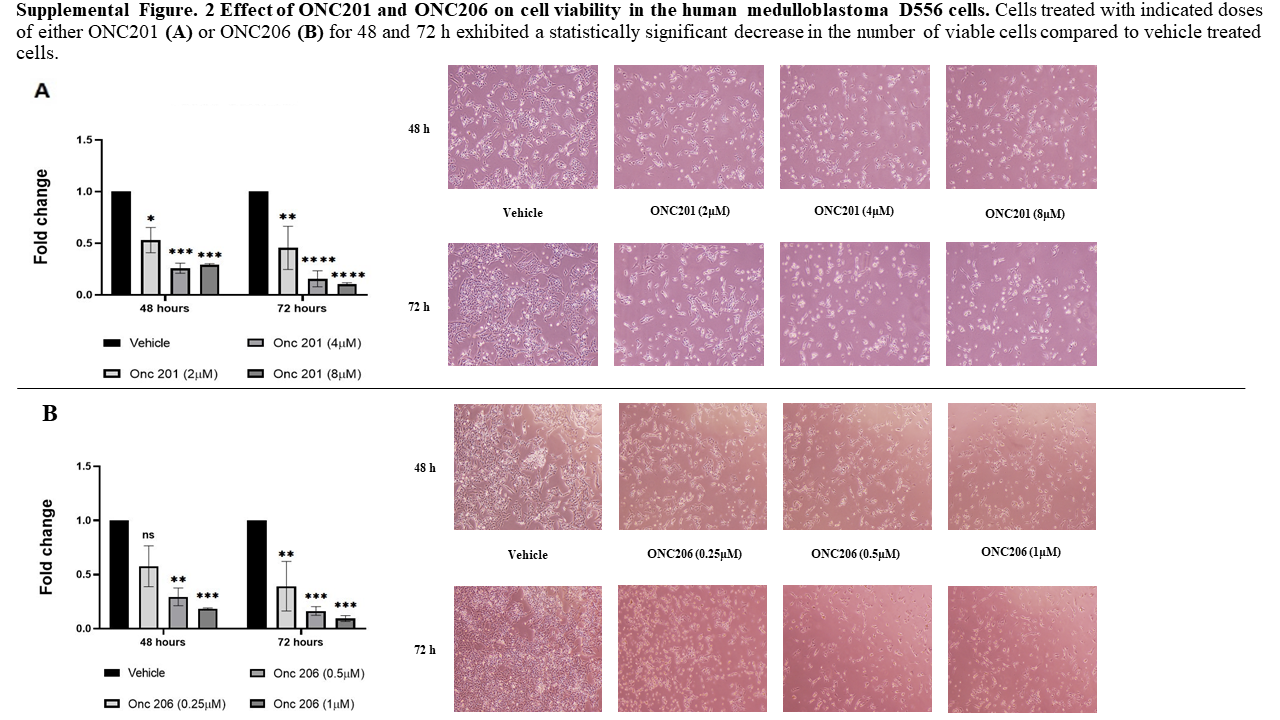

Supplement: Supplementary file 2 [file Image_2.TIF]

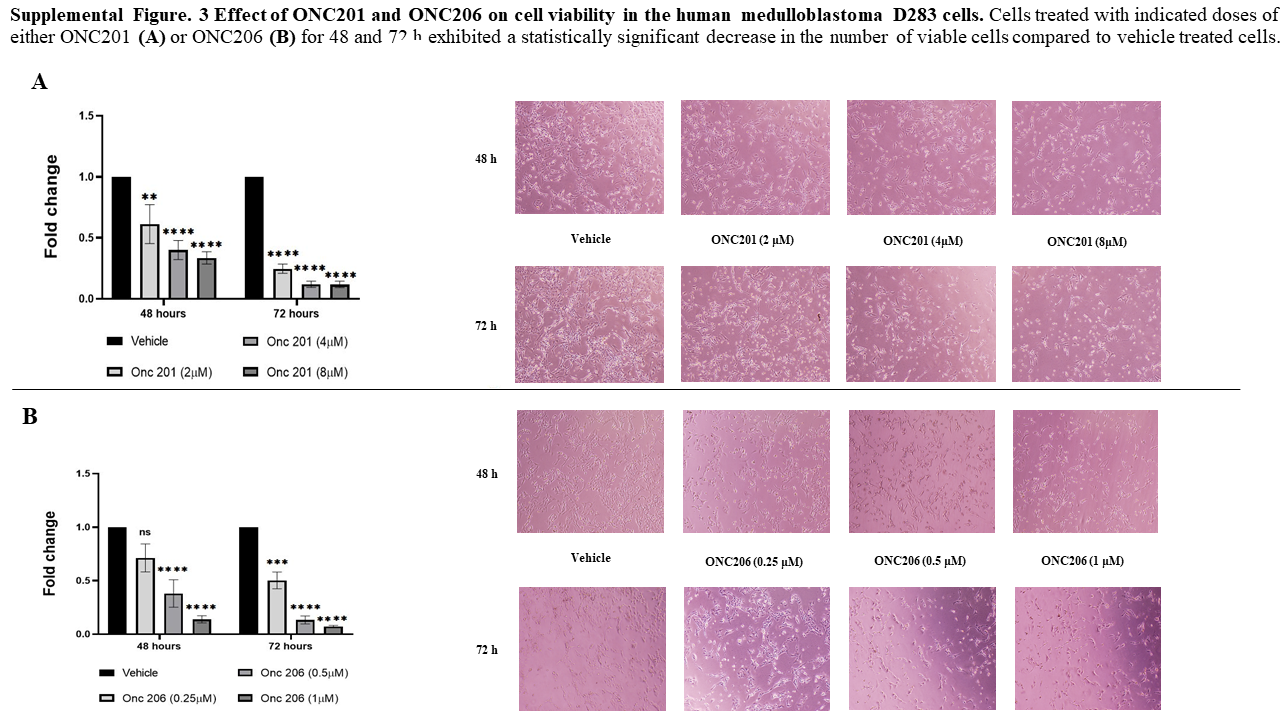

Supplement: Supplementary file 3 [file Image_3.TIF]

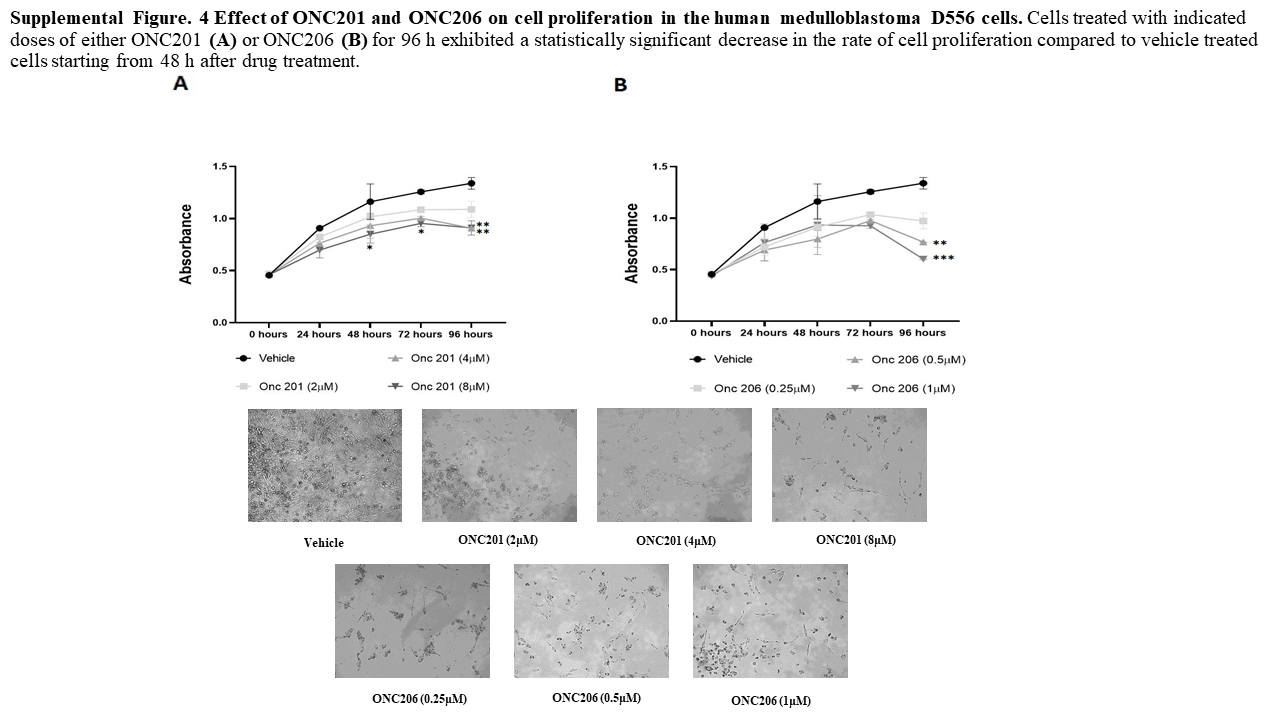

Supplement: Supplementary file 4 [file Image_4.TIF]

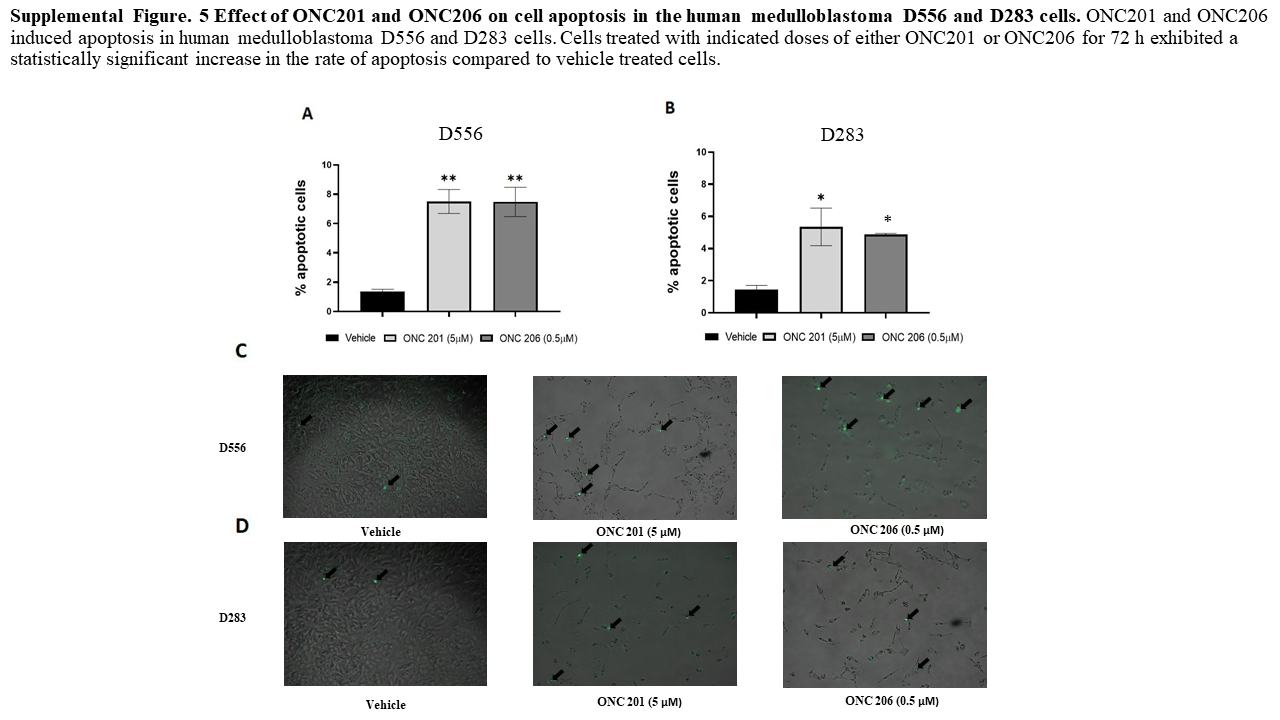

Supplement: Supplementary file 5 [file Image_5.TIF]

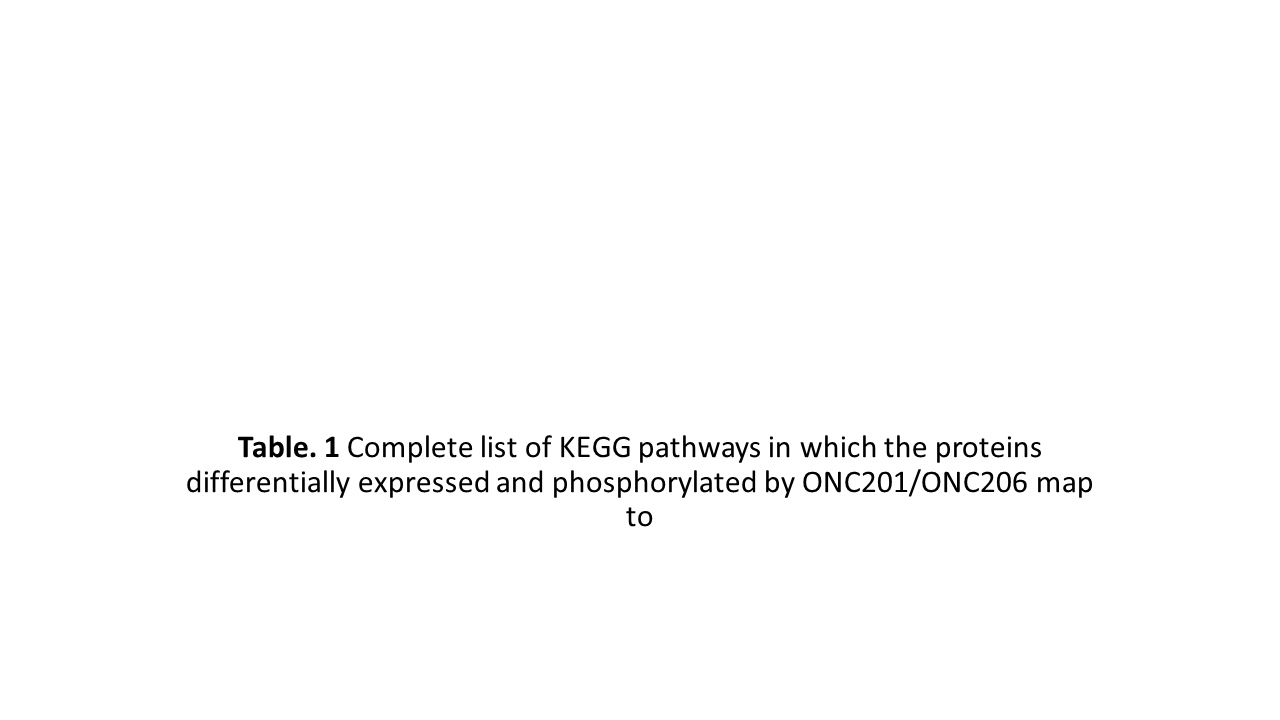

Supplement: Supplementary file 6 [file Image_6.TIF]

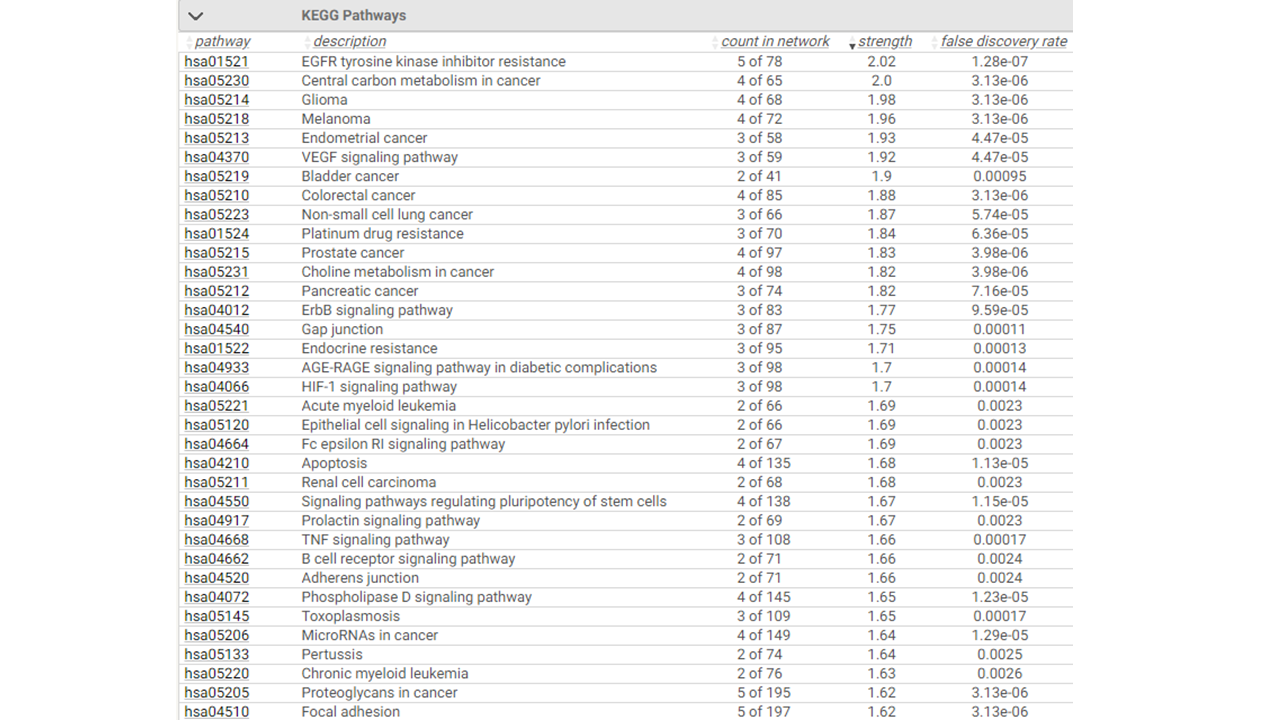

Supplement: Supplementary file 7 [file Image_7.TIF]

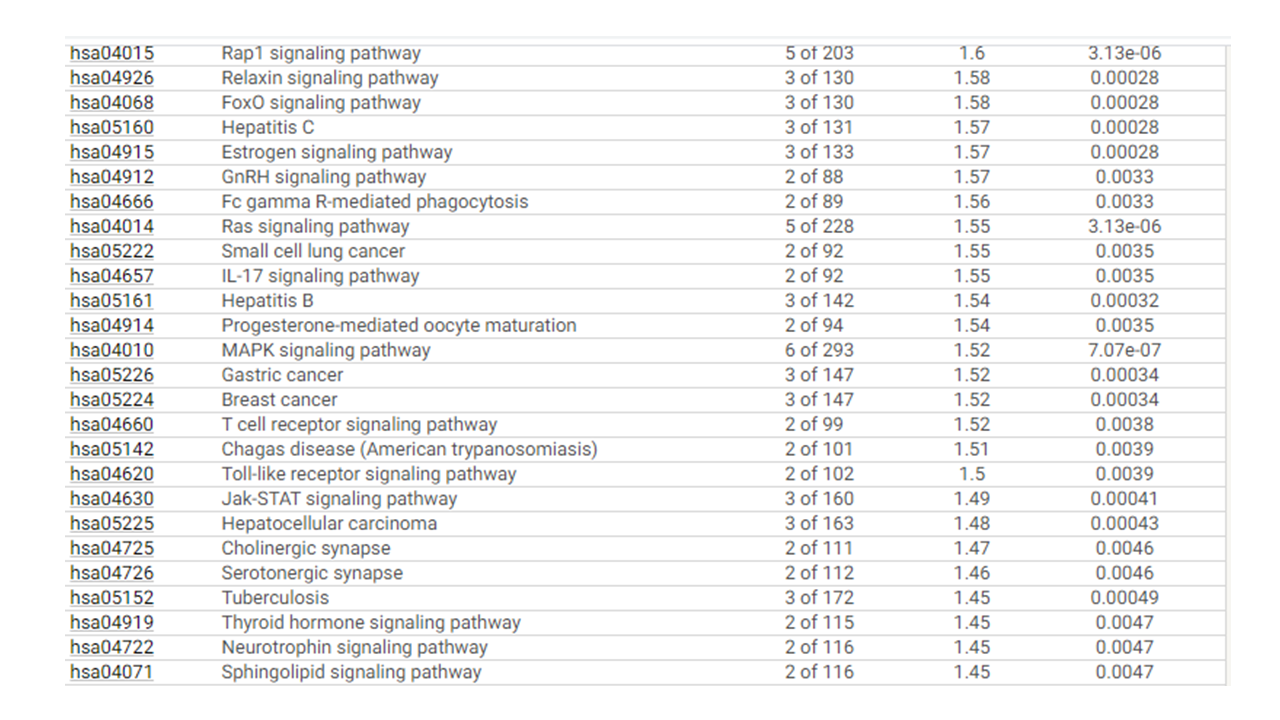

Supplement: Supplementary file 8 [file Image_8.TIF]

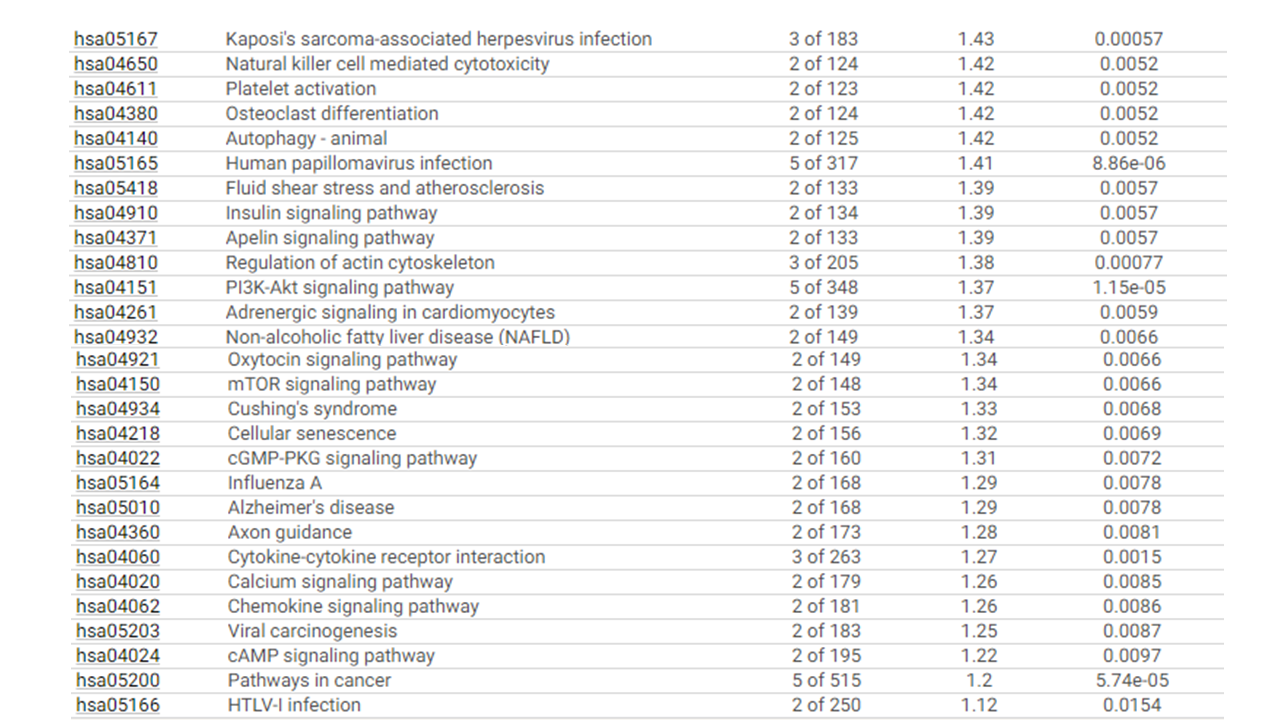

Supplement: Supplementary file 9 [file Image_9.TIF]
